# Elimination of “kitome” and “splashome” contamination results in lack of detection of a unique placental microbiome

**DOI:** 10.1186/s12866-020-01839-y

**Published:** 2020-06-11

**Authors:** Isoken Nicholas Olomu, Luis Carlos Pena-Cortes, Robert A. Long, Arpita Vyas, Olha Krichevskiy, Ryan Luellwitz, Pallavi Singh, Martha H. Mulks

**Affiliations:** 1 Department of Pediatrics & Human Development, Division of Neonatology, Michigan State University, East Lansing, MI USA; 2 Facultad de Ciencias Agrarias, Universidad de Pamplona, Pamplona, Colombia; 3 Department of Obstetrics & Gynecology, Sparrow Hospital, Lansing, MI USA; 4 Department of Obstetrics & Gynecology, Michigan State University, East Lansing, MI USA; 5 Department of Pediatric Endocrinology, California North State University, Elk Grove, CA USA; 6 Department of Obstetrics & Gynecology, SSM Health/Dean Medical Group, Madison, WI USA; 7 Department of Biological Sciences, Northern Illinois University, DeKalb, IL USA; 8 Department of Microbiology & Molecular Genetics, Michigan State University, East Lansing, MI USA

**Keywords:** Placenta, Microbiome, Kits, Reagents, ‘Splashome’, Contaminants

## Abstract

**Background:**

A placental microbiome, which may be altered in gestational diabetes mellitus (GDM), has been described. However, publications raising doubts about the existence of a placental microbiome that is different than contaminants in DNA extraction kits and reagents (“kitomes”) have emerged. The aims of this study were to confirm the existence of a placental microbiome distinct from contaminants and determine if it is altered in GDM mothers.

**Results:**

We first enrolled normal weight, obese and GDM mothers (*N* = 17) at term elective cesarean section delivery in a pilot case control study. Bacterial DNA was extracted from placental parenchyma, maternal and cord blood, maternal vaginal-rectal swabs, and positive and negative controls with the standard Qiagen/MoBio Power Soil kit. Placentas had significantly higher copies of bacterial 16S rRNA genes than negative controls, but the placental microbiome was similar in all three groups and could not be distinguished from contaminants in blank controls. To determine the source and composition of the putative placental bacterial community identified in the pilot study, we expanded the study to 10 subjects per group (*N* = 30) and increased the number and variety of negative controls (*N* = 53). We modified our protocol to use an ultraclean DNA extraction kit (Qiagen QIAamp UCP with Pathogen Lysis Tube S), which reduced the “kitome” contamination, but we were still unable to distinguish a placental microbiome from contaminants in negative controls. We noted microbial DNA from the high biomass vaginal-rectal swabs and positive controls in placental and negative control samples and determined that this resulted from close proximity well-to-well cross contamination or “splashome”. We eliminated this source of contamination by repeating the sequencing run with a minimum of four wells separating high biomass from low biomass samples. This reduced the reads of bacterial 16S rRNA genes in placental samples to insignificant numbers.

**Conclusions:**

We identified the problem of well-to-well contamination (“splashome”) as an additional source of error in microbiome studies of low biomass samples and found a method of eliminating it. Once “kitome” and “splashome” contaminants were eliminated, we were unable to identify a unique placental microbiome.

## Background

The Human Microbiome Project (HMP) was initiated to characterize and compare the complex microbial communities that inhabit different niches of the healthy adult human body, including the skin, nasal passages, oral cavity, gastrointestinal tract, and urogenital tract in an attempt determine whether a core healthy human microbiome exists in each of these sites [1, 2]. This project has generated an extensive database using sequencing of bacterial 16S rRNA genes. By comparing extracted 16S rRNA gene results to historic HMP data, Aagaard et al. [3], published the existence of a unique placental microbiome that was described as most comparable to the oral microbiome. The placental samples used in that study were from diverse sources, including term and preterm pregnancies and vaginal and cesarean section deliveries, and included mothers with remote history of infection during the pregnancy [3].

Subsequently, there have been reports of the possible involvement of the placenta in fetal macrosomia in gestational diabetes mellitus (GDM) [4], an altered placental microbiome in pregnancies complicated by GDM [5, 6] and of correlations between the placental microbiome and fetal macrosomia in mothers with GDM [7]. These studies are of particular interest given extensive data from humans and laboratory animals indicating obesity and insulin resistance are associated with alterations in the “normal” gastrointestinal microbiome [8, 9], and suggestions there is an “obesity-associated” gut microbiome [10, 11]. We embarked on this study to investigate the possible existence of a “macrosomia-associated” placental microbiome in mothers with GDM.

In designing our investigation, we acknowledged and took heed of the growing volume of published data refuting the existence of a unique placental microbiome, calling into question the methods and controls of the above studies, all of which lack positive and negative controls [12–15]. We were mindful of likely sources of error and attempted to control for potential contamination wherever possible. We recruited patients equally into three groups: normal weight (control), obese (control), and GDM. To limit contamination by rectovaginal microorganisms or concurrent infection, our enrolled study and control mothers were all delivered by scheduled cesarean section at term gestation, without labor and with intact fetal membranes. We also took cognizance of criticisms leveled at the initial description of the placental microbiome by including maternal and fetal blood specimens to control for organisms that may be present in the bloodstream (so called ‘dormant blood microbiome’ [16]) that may seed the placenta [17]. We further included a vaginal-rectal (VR) swab (high biomass sample) obtained from each mother to serve as a positive control. More importantly, several studies had drawn attention to microbial DNA contamination of laboratory reagents [18], DNA extraction kits [19] and other laboratory and clinical equipment [20, 21] that could interfere with interpretation of data from microbiome studies, especially of tissues with low bacterial biomass like the placenta. Such contaminants have included water-borne bacterial genera (*Pseudomonas, Stenotrophomonas, Xanthomonas, Ralstonia* and *Bacillus*) [18], and soil and plant associated bacteria (*Sphingobacteriaceae, Bradyrhizobiaceae, Methylobacterium* and *Phyllobacteriaceae*) [12, 21, 22]. Several of these bacteria have been commonly reported as part of the placental microbiome [3, 23–25]. We therefore included in our study design multiple negative or blank controls. These included NH_4_Cl, 80% EtOH, ‘sterile’ swabs, ‘sterile’ swabs exposed to operating room or sampling room air as well as used to sample surfaces, extraction reagents alone and reagents run through the kits.

The problem of bacterial DNA contamination of reagents, extraction kits and air in the delivery room was evident in the pilot data from our study of placentas from 17 subjects that included five normal weight, six obese and six gestational diabetic mothers [26]. Our pilot study utilized the standard Qiagen/MoBio PowerSoil DNA extraction kit recommended by the HMP and was unable to differentiate a placental low biomass microbiome that was distinct from the contaminants in blanks or negative controls [26]. The subsequent expanded study reported here, which included 30 subjects, was designed to eliminate as much extraction kit contamination (“kitome”) as possible, utilizing the new ultraclean Qiagen QIAamp UCP Mini Kit with Pathogen Lysis Tube S DNA extraction kit with additional negative and positive controls. While this method greatly reduced potential contamination from the DNA extraction process, we identified another source of contamination that we have termed the “splashome” (well-to-well contamination during sequencing plate preparation and run).

Our overall hypothesis was that gestational diabetes is associated with alterations in the placental microbial community which may provide explanations for macrosomia observed in some infants of gestational diabetic mothers. Further, we wanted to demonstrate the presence of a placental microbiome that is different from reagent and kit contaminants. Results from the expanded study however showed that once the “kitome” and “splashome” sources of contamination were removed, we could not identify a placental microbiome distinguishable from contaminants in blank controls.

## Results

### Characteristics of mothers and infants in the expanded study

Characteristics of the study subjects in the expanded study are shown in Table 1. Subjects with GDM were significantly older than obese and normal weight mothers but with similar first trimester and third trimester BMIs as the obese mothers. Obese and GDM mothers had significantly greater BMIs than normal weight mothers. Obese and normal weight mothers had normal 1-h glucose tolerance test (GTT) results compared to the GDM mothers. The three groups had similar rates of Group B *Streptococcus* (GBS) colonization and their infants had similar birth weights. Of the mothers with GDM, 6 were diet controlled, 3 were on insulin and one was managed with Metformin. Four infants were admitted to the neonatal intensive care unit: two infants of obese mothers and one of normal weight mothers for transient tachypnea of the newborn, and one infant of a GDM mother for hypoglycemia.

**Table 1:** Characteristics of Mothers and Infants in the Expanded Study

Maternal & Infant Characteristics	GDM (***N*** = 10)	Obese (***N*** = 10)	Normal Weight (***N*** = 10)	***ρ***-Value
Maternal age [yrs, mean (SD)]	34.2 (5.5)	26.6 (5.0)	30.3 (3.9)	< 0.005
1st Trimester BMI (kg/M^2^, median)	33.9	33.8	22.9	< 0.001
3rd Trimester BMI (kg/M^2^, median)	36.9	36.9	26.4	< 0.001
GBS Positive (%)	20	40	40	N/S
1 h GTT, Mean (SD)	159.3 (15.5)	103.5 (16.3)	98.1 (23.2)	< 0.001
Infant birth weight (kg)	3.46 (0.47)	3.59 (0.45)	3.23 (0.42)	N/S

*GDM* Gestational diabetes mellitus, *SD* standard deviation, *GBS* Group B *Streptococcus, GTT* glucose tolerance test, *N/S* not significant. Statistical analysis was performed with SigmaPlot V14.0, San Jose CA

### Pilot study

#### Quantitation of bacterial biomass in patient samples and controls using real time qPCR

**Fig. 1:**
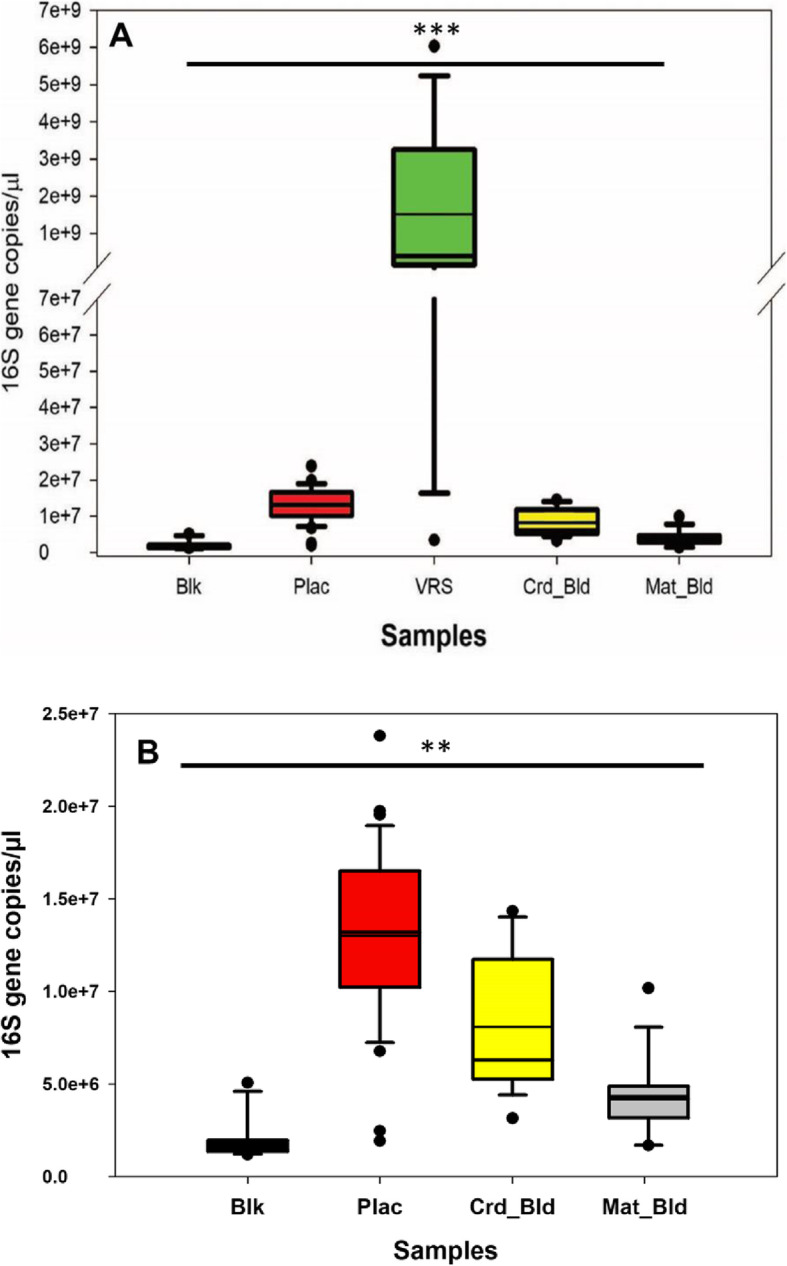
Quantitative PCR of bacterial 16S rRNA gene abundance in pilot study samples and blank controls. Quantitative PCR was performed on DNA extracts from blank controls (Blk; *N* = 13) placenta (Plac; *N* = 35), vagino-rectal swabs (VRS; *N* = 16), cord blood (Crd bld; *N* = 16), and maternal blood (Mat bld; *N* = 16) from the pilot study. **a** VRS samples were significantly (*ρ* < 0.0001, ***) different from placenta, maternal and cord blood, and blank controls as measured by ANOVA followed by Tukey’s test. **b** Analysis of the low biomass samples in the absence of the very high biomass VRS samples, using ANOVA followed by Tukey’s, showed that placental samples had a significantly higher bacterial biomass than the other samples (*ρ* < 0.01, **)

Quantitative PCR of bacterial 16S rRNA genes in the study samples and blank controls was used to determine relative absolute values of bacterial DNA in these samples (Fig. 1a and b). VR samples overall contained a much higher biomass (*ρ* < 0.0001), based on concentrations of bacterial 16S rRNA genes, compared to placenta, maternal and cord blood samples, and blank controls (Fig. 1a). While placental samples contained a very low biomass of bacteria, the average copy number of bacterial 16S rRNA genes was significantly higher in placental samples than in either maternal blood (*ρ* < 0.001) or cord blood samples (*ρ* < 0.01) or blank controls (*ρ* < 0.001) (Fig. 1b). Cord blood samples contained higher biomass than blanks (*ρ* < 0.001) and maternal blood (*ρ* < 0.05). In contrast, maternal blood samples did not differ from blank controls.

#### 16S rRNA gene surveys of bacterial communities in patient VR samples and controls from the pilot study

Sequencing of 16S rRNA gene libraries of this set of samples showed that the high biomass VR samples overall contained bacteria representative of known vaginal and rectal communities. VR communities from all three groups were dominated by members of the phylum Firmicutes, with Actinobacteria and Bacteroidetes also abundant (Supplemental Fig. S1). Small numbers of Fusobacteria, Proteobacteria, Tenericutes, and Verrucomicrobia were also found.

At the family level (Supplemental Fig. S2), VR communities contained primarily *Lactobacillaceae* (*Lactobacillus* species), which are normal vaginal microbiota, and Clostridiales, particularly *Tissierellaceae* and/or *Ruminococcaceae*, which are normal fecal microbiota, as well as Bacteroidetes *(Prevotellaceae and Porphyromonadaceae*). VR samples from normal BMI patients contained predominantly Lactobacillaceae, while those from obese patients contained more Clostridiales and Bacteroidetes and those from GDM patients were varied. Overall, particularly with this small sample size, no statistical difference was observed between VR samples from the 3 study groups by either PERMANOVA (Bray-Curtis *ρ* = 0.2137 and Jaccard *ρ* = 0.6612) or by ANOSIM (Bray-Curtis *ρ* = 0.3145 and Jaccard *ρ* = 0.6905).

#### 16S rRNA gene surveys of bacterial communities in patient placental samples and controls from the pilot study

Sequencing of 16S rRNA gene libraries of the placental samples showed strong similarity, at the phylum level, in the three study groups, although there was variability between patients within groups (Supplemental Fig. S3). This microbiome was dominated by the phyla Proteobacteria and Firmicutes, with smaller amounts of Actinobacteria and Bacteroidetes. It is noteworthy that the distribution of phyla seen in the blank controls was very similar to that in the placental samples, although there were larger numbers of Firmicutes and fewer Proteobacteria in the blanks.

At the family level, (Supplemental Fig. S4) overall, members of the phylum Proteobacteria dominated the placental microbiomes of all three study groups, with Caulobacteriaceae (*Caulobacter*), Methylobacteriaceae (*Methylobacterium*), and Oxalobacteraceae (*Ralstonia*) most frequently seen. Members of the Firmicutes, primarily Clostridiales families Lachnospiraceae, Ruminococcaceae, and Tissierellaceae, were also seen in large numbers. Organisms potentially of oral origin, such as *Prevotellaceae* and *Streptococcaceae,* were found but these were minor components of the placental findings in our study. *Oxalobacteraceae* (*Ralstonia* spp.) was the only organism found in all placental samples, representing ~ 14% of the total placental ‘microbiome’. *Caulobacter, Methylobacterium, Sphingomonas,* and *Acinetobacter* were found in most blank controls and most placental samples. Further, organisms that were common in the VR samples, such as *Lactobacillaceae, Tissierellaceae,* and *Enterobacteriaceae*, were also seen in many placental samples but also in many blank controls. Although the qPCR data suggested that placental samples contained a higher biomass of 16S rRNA genes than the blank controls, we were unable to clearly differentiate placental samples from blanks with these data. No statistical difference was observed between placental samples from the 3 study groups by either PERMANOVA (Bray-Curtis *ρ* = 0.2954 and Jaccard *ρ* = 0.6911) or ANOSIM (Bray-Curtis *ρ* = 0.2799 and Jaccard *ρ* = 0.74). Further, no statistical difference was observed between the total placental samples and blanks by either PERMANOVA (*ρ* = 0.103) or by ANOSIM (*ρ* = 1).

### Expanded study

We expanded the study to a larger cohort of 10 subjects per group because, although we detected more bacterial DNA in placental samples, we were unable to distinguish the placental microbial community in the three study groups from contaminants in the kit and reagent blanks. Furthermore, we chose a new ultraclean DNA extraction kit (Qiagen QIAamp UCP Pathogen Mini Kit with Pathogen Lysis Tube S) to reduce contamination of the microbiome data. Controls included all previous controls such as swabs, and all reagents, both alone and processed through the extraction kits, as well as sequencing reaction controls and positive controls.

#### Quantitation of bacterial biomass in patient samples and controls using real time qPCR

**Fig. 2:**
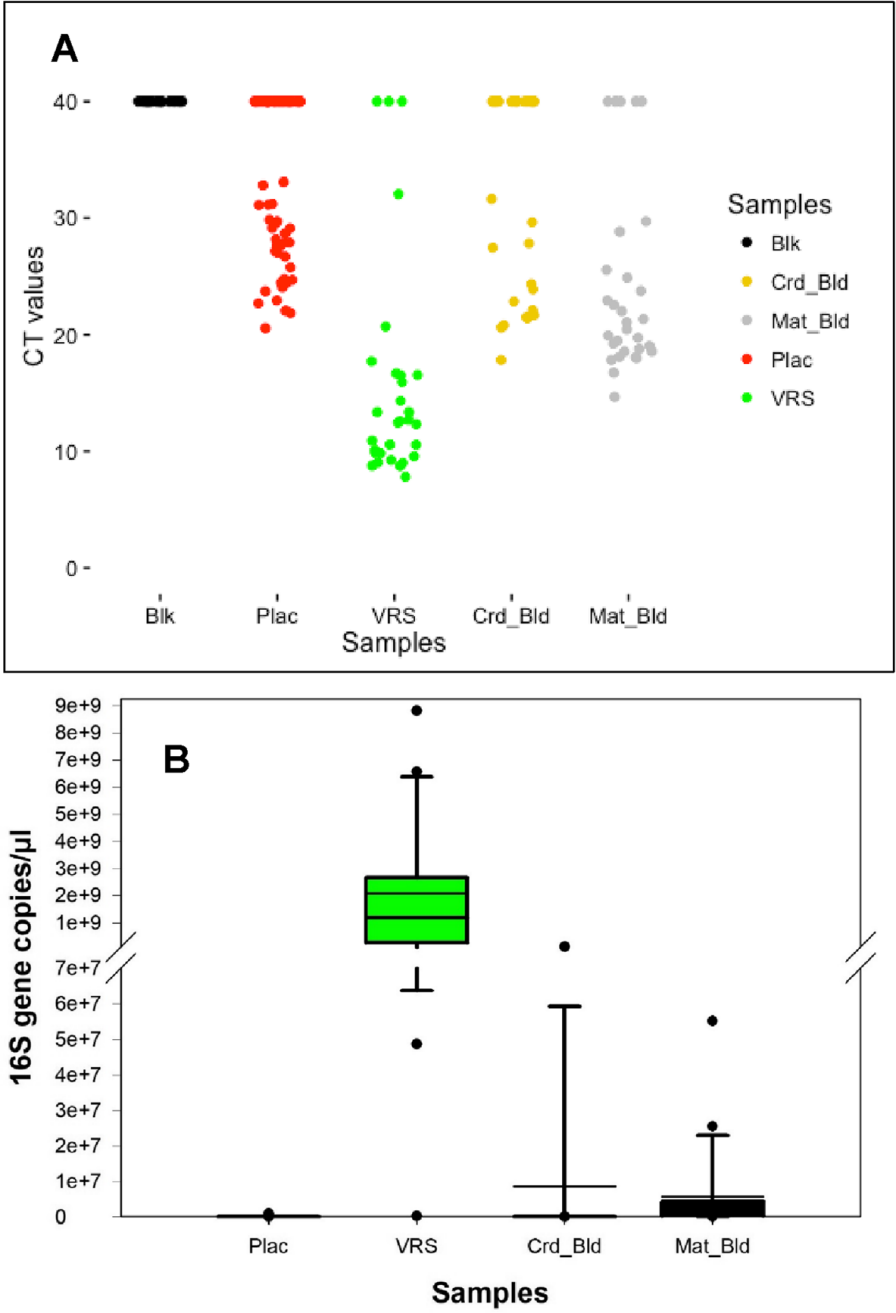
Quantitative PCR of bacterial 16S rRNA gene abundance in expanded study samples and blank controls. Quantitative PCR was performed on DNA extracts from blank controls (Blk = 53), placenta (Plac; *N* = 60), vagino-rectal swabs (VRS; *N* = 28), cord blood (Crd bld; *N* = 28), and maternal blood (Mat bld; *N* = 28) from the expanded study. **a** CT value represented for individual samples from the various sites including blanks. Undetermined and/or low confidence Cq CT values, i.e., below the limit of reliable detection (CT > 35), are plotted with hypothetical value of 40. **b** Box plot of estimated 16S rRNA gene copy number for samples with CT values less than 35, including placenta (Plac; *N* = 31 of 60 total samples), vagino-rectal swabs (VRS; *N* = 27/28), cord blood (Crd bld; *N* = 14/28), and maternal blood (Mat bld; *N* = 25/28) from the expanded study. Undetermined and/or low confidence Cq CT values, i.e., below the limit of reliable detection (CT > 35), for some samples and all 53 blanks contributed to discrepancy in the frequency of samples plotted as well as omission of blanks from the plot. VRS were significantly (*** *ρ* < 0.0001) different from Plac, Crd_Bld, and Mat_Bld (ANOVA followed by Tukey’s test). However, placenta, cord blood, and maternal blood samples were not significantly different (*ρ* = 1)

As in the pilot study, quantitative PCR of bacterial 16S rRNA genes was used to determine relative absolute values of bacterial DNA in the placenta, VR swabs, maternal blood, cord blood, and blank control samples collected for the expanded study (Fig. 2a and b). VR samples overall contained a similar biomass, based on concentrations of bacterial 16S rRNA genes, as seen in VR samples from the pilot study. However, many of the placental, maternal blood, and cord blood samples, and all of the blank control samples, contained levels of bacterial 16S rRNA gene that were below the limit of accurate detection in the assay. The placenta, maternal blood, and cord blood samples that could be analyzed were not statistically different from each other.

### 16S rRNA gene surveys of bacterial communities in patient VR samples and controls from the expanded study

**Fig. 3:**
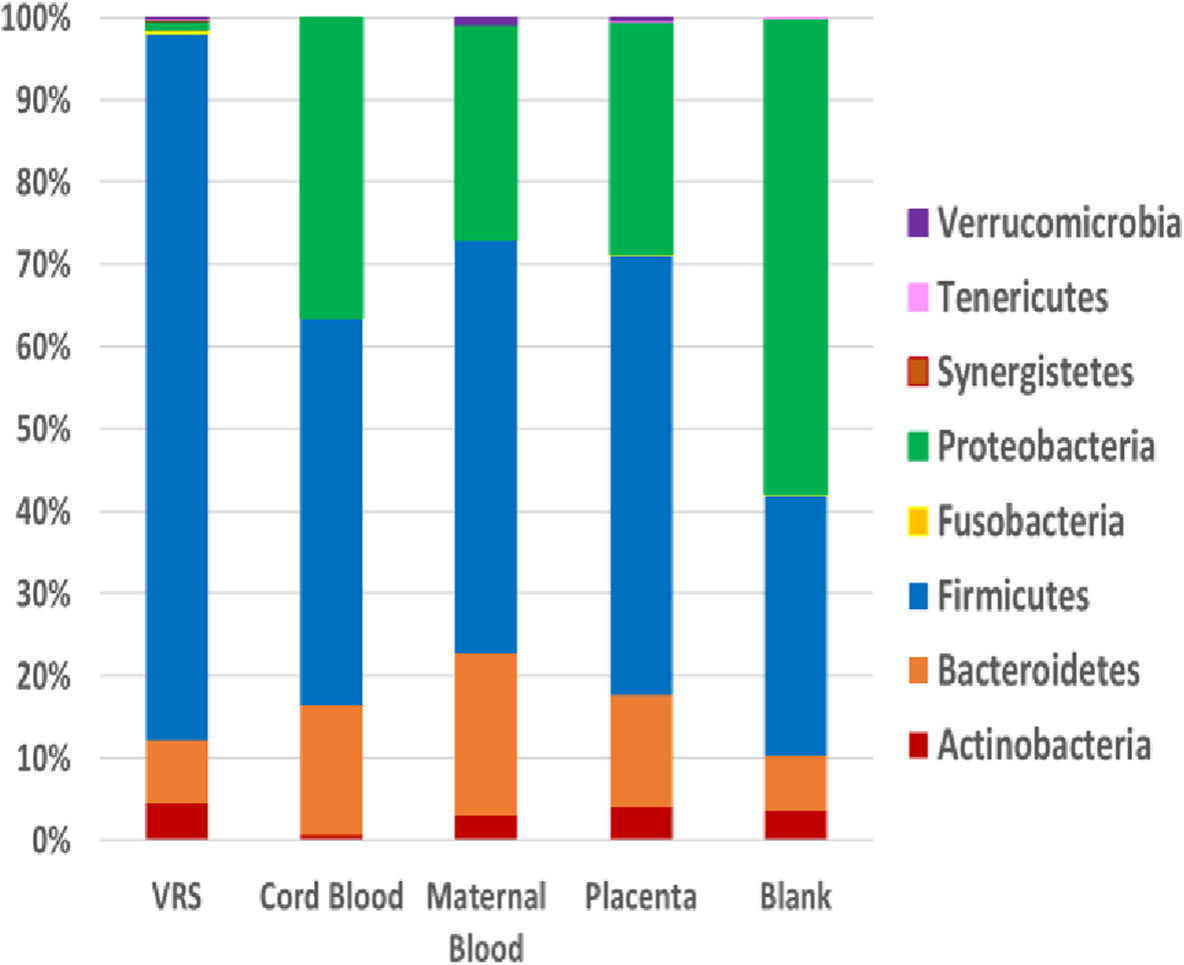
The microbial communities at the phylum level in all samples from the expanded study. Colored bars illustrate the percentage of total reads classified into specific phyla and are the average of all samples from all three study groups and all blanks. Average number of reads for each sample type: VRS, 180759; cord blood, 26; maternal blood, 36, placenta, 59; and blanks, 100

At the phylum level, VR samples from the expanded study were very similar to those from the pilot study and contained predominantly Firmicutes (~ 86%) and very few Proteobacteria (Fig. 3). There were ~ 180,000 quality filtered final reads on average for the VR samples. In contrast, placental, maternal blood, and cord blood samples from the expanded study contained on average fewer than 100 reads per sample, which was similar to the average number of reads in the blanks. While this number of reads is too low to be statistically accurate, we did analyze these samples and found proportionately fewer Firmicutes and proportionately more Proteobacteria than VR samples, and the relative amount of Proteobacteria was lower in these samples than was seen in the pilot study. Blanks contained mainly Proteobacteria with fewer Firmicutes. All samples contained small amounts of Actinobacteria and Bacteroidetes.

**Fig. 4:**
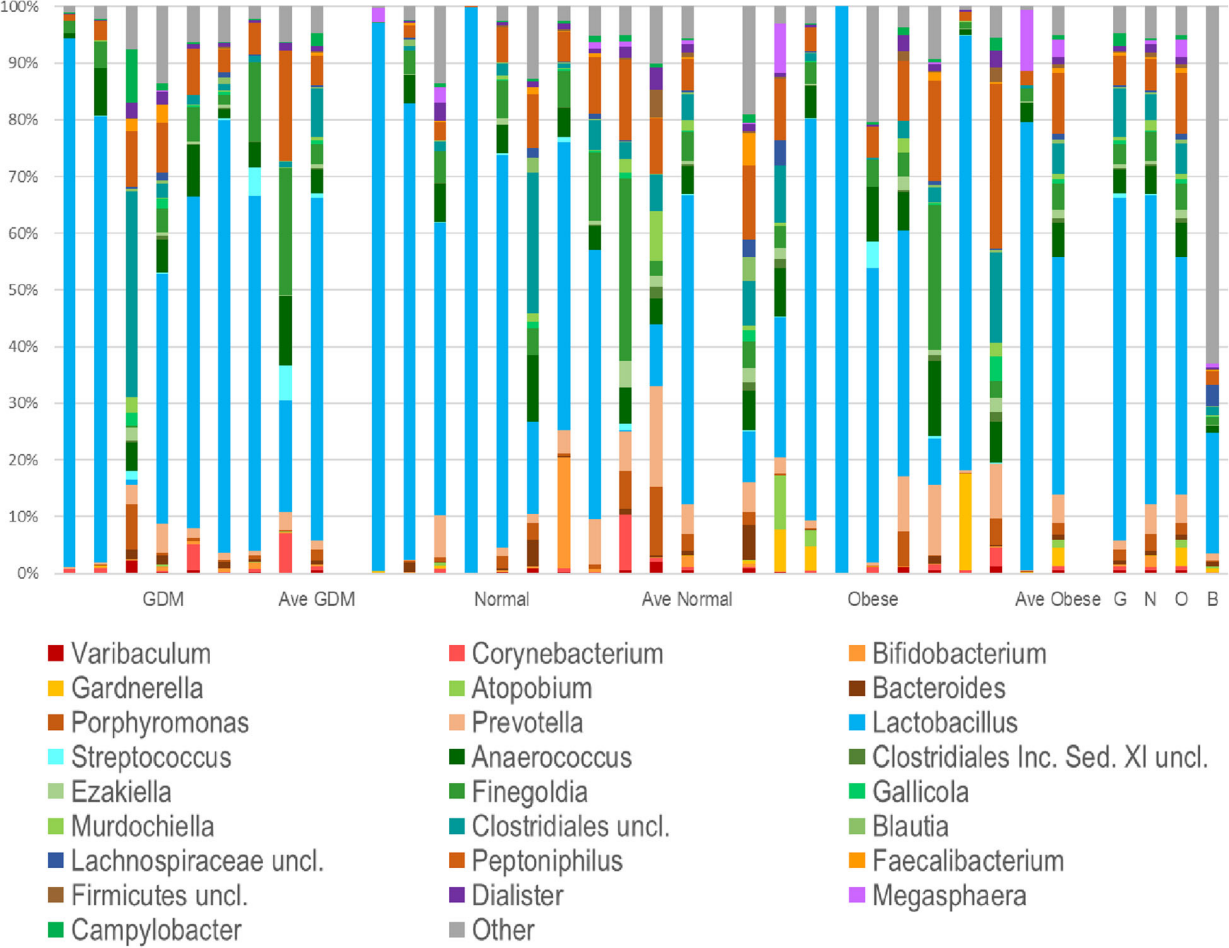
The VR microbial community at the genus level from the expanded study samples. The top 25 genera (of 105 total identified) found in VRS communities are shown. Colored bars illustrate the percentage of total reads classified into specific genera. The last column in each group shows the average for that set of patients. These average columns are repeated on the far right for ease of comparison, and are labeled G = GDM, N = Normal, O = Obese, and B = Blanks. Uncl. = unclassified; Inc. Sed. = Incertae Sedis

In this expanded study, we were able to classify ~ 90% of reads to the genus level (Fig. 4). *Lactobacillus* was the most common genus seen in almost all samples, representing on average 40–60% of all reads, although there was extensive patient-to-patient variation. The three most common OTUs, which varied in distribution among patients, were identified as belonging to this genus. Members of the order Clostridiales, particularly *Peptoniphilus, Anaerococcus, Finegoldia* and unclassified Clostridiales, which are common fecal organisms, together represented ~ 25% of reads in the normal BMI and GDM patients, and ~ 33% in obese patients, although there was extensive patient-to patient-variation in the distribution of these genera. Genera belonging to the phyla Actinobacteria and Bacteroidia were seen in smaller numbers in most patients. *Campylobacter (Campylobacteriaceae)* were seen in small numbers in most patients, but *Enterobacteriaceae* were rare. As seen in the pilot study, a few patients had increased Bacteroidetes (*Prevotella* and *Porphyromonas*) although this did not correlate with any specific study group. We did note that three genera, *Gardnerella, Atopobium,* and *Megasphaera,* which are associated with bacterial vaginosis, were found more frequently in VR samples from obese patients, although only in 4 of the 10 patients.

**Fig. 5:**
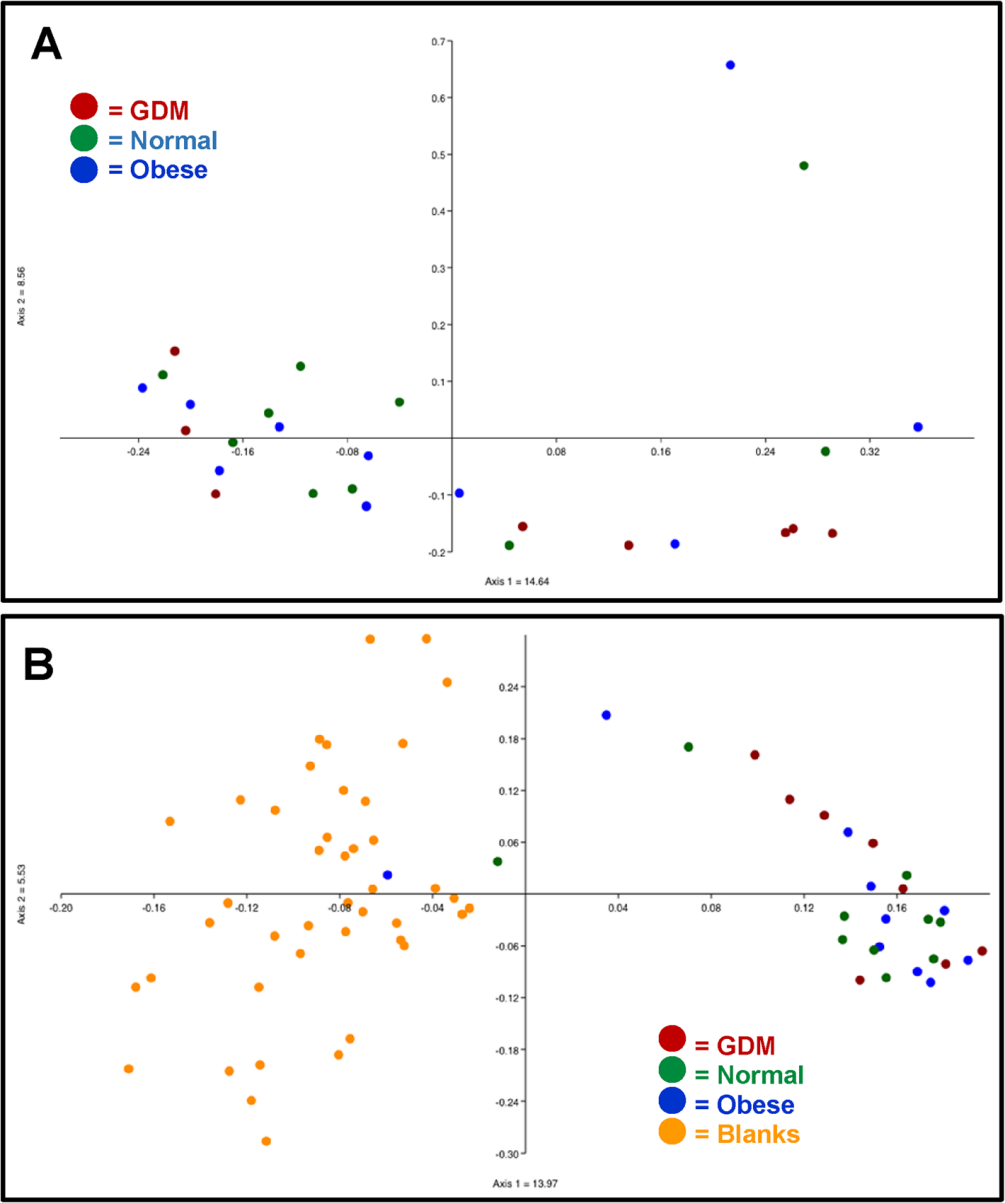
Principal coordinates analysis (PCoA) of VR samples from the expanded study. **a** VR samples do not cluster by study group. PCoA based on Jaccard distances is shown. **b** VR samples segregate from blank controls. PCoA based on Jaccard distances is shown

We asked whether the VR communities clustered by study group and found that they did not by either Jaccard analysis (Fig. 5a), which measures sample composition, e.g. presence/absence of community members, or by Bray-Curtis analysis, (data not shown), which measures sample structure, e.g., presence/absence and abundance. No statistical difference was observed between VR communities clustered by study group as analyzed by ANOSIM (Bray-Curtis *ρ* = 0.9457 and Jaccard *ρ* = 0.1092). However, these communities did cluster by the specific OTU of *Lactobacillus*, of three most abundant OTUs seen*,* that predominated in each sample (data not shown). The VR communities did clearly segregate from the communities found in blank controls (Fig. 5b), and this was statistically significant as analyzed by ANOSIM (Bray-Curtis *ρ* = 0.0085 and Jaccard *ρ* = 0.0001).

### 16S rRNA gene surveys of bacterial communities in patient placental samples and controls from the expanded study

**Fig. 6:**
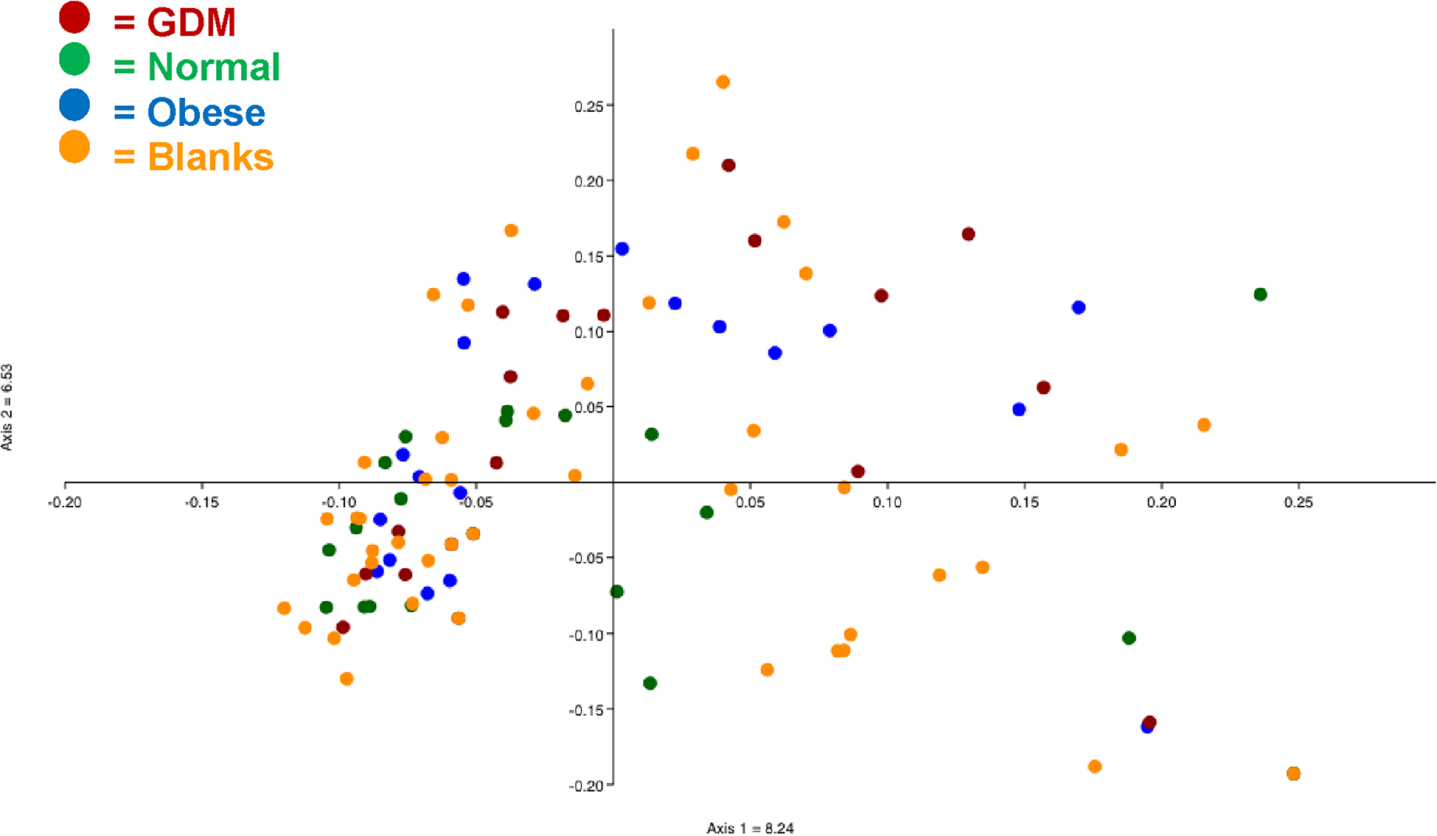
Principal coordinates analysis (PCoA) of placental samples from the expanded study. Placental samples did not segregate by study group and did not segregate from blank controls. PCoA analysis based on Jaccard distances is shown

DNA was extracted from two separate placental samples (P1 and P2) randomly selected from each patient and sequencing of the 16S rRNA genes performed. We found that the placental samples on average had very low numbers of reads, similar to blank controls, as did the maternal blood and cord blood samples (Supplemental Fig. S5). Overall, due in part to the very low reads in these samples, we could not distinguish samples from GDM, normal BMI, and obese patients. Placental samples did not segregate by study group and did not segregate from blank controls (Fig. 6); this was supported by ANOSIM analysis (Bray-Curtis *ρ* = 0.4495 and Jaccard *ρ* = 0.7907).

**Fig. 7:**
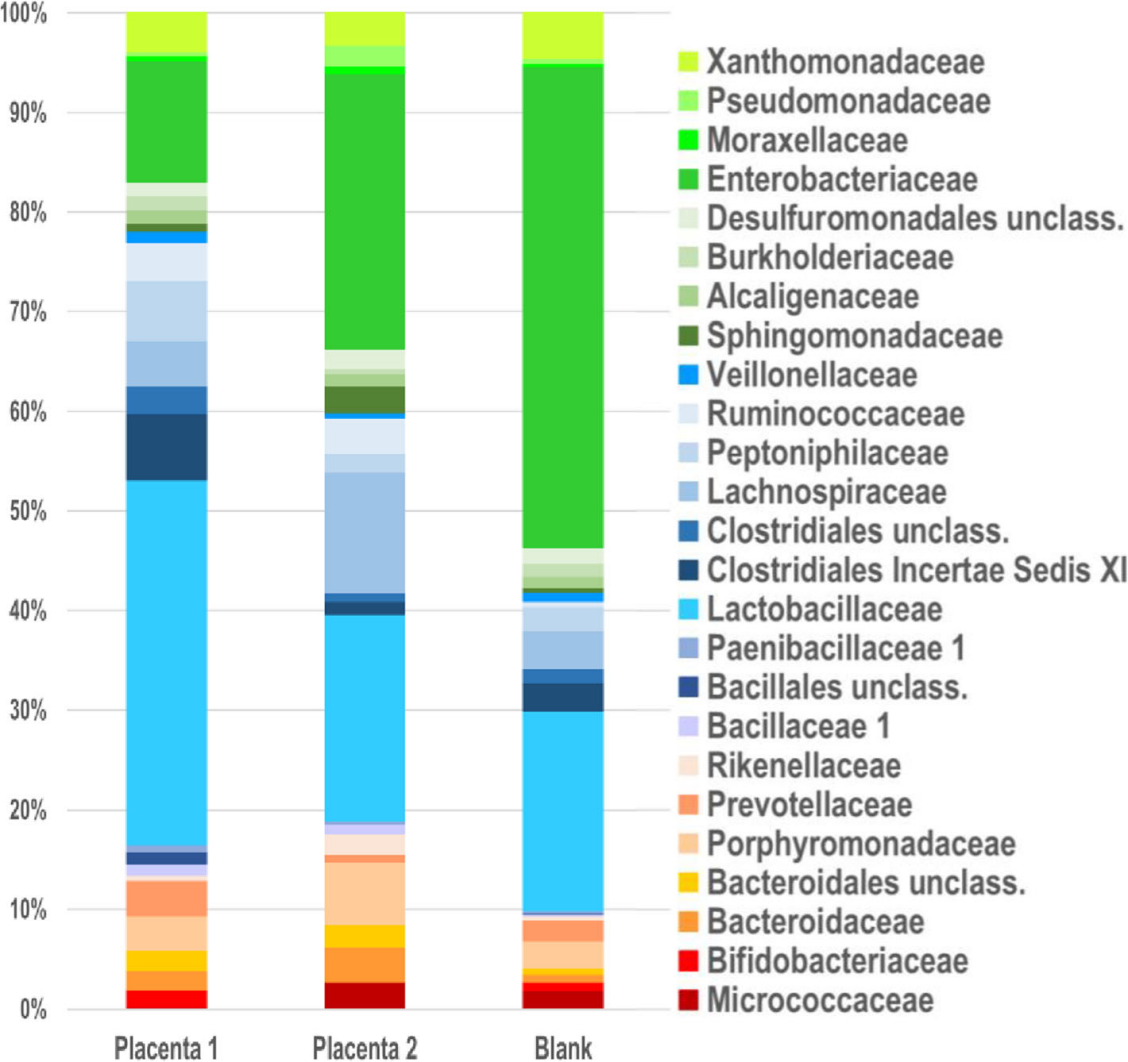
The microbial community at the family level in two separate placental samples (P1 and P2) and blanks from the expanded study. The top 25 families (of 90 total identified) found in these communities are shown. Each column shows the sum of all samples in that group, i.e., 30 P1 and 30 P2 samples (10 each from GDM, normal BMI and obese groups) and 53 separate blank controls. Total reads for each set of samples includes 2183 P1, 835 P2, and 4467 blanks. Colored bars illustrate the percentage of total reads represented by each family shown. Placenta 1 (P1) and placenta 2 (P2) represent two different placental samples from each patient

OTUs found in placental samples often included organisms common in the VR samples, such as *Lactobacillus, Prevotella*, *Peptoniphilus*, *Ruminococcus*, and other Clostridiales, but were also overall very similar to blank controls (Figs. 6 and 7). We considered that the placenta could have acquired VR organisms by ascension through the cervix, although not during the delivery process as all patients were delivered by scheduled Cesarean section without labor. However, we found a discrepancy between P1 and P2 samples (Fig. 7, Table 2). P1 samples on average had 2.7-fold more reads compared to P2 samples, and P1 samples also were more similar to VR samples than P2 samples were. For example, P1 samples contained higher amounts of Lactobacillaceae, Clostridiales Incertae Sedis XI, Peptoniphilaceae, Ruminococcaceae, and Prevotellaceae, which are common components of the VR microbiome (Fig. 4), than did P2 samples (Table 2).

**Table 2:** Distribution of bacterial families between P1 and P2 samples and blanks

Family^**a**^	Placenta 1 (***n*** = 30)	Ave P1	Placenta 2 (***n*** = 30)	Ave P2	Blank (***n*** = 46)	Ave Blank
*Enterobacteriaceae*	267	8.9	231	7.7	2023	44.0
***Lactobacillaceae***	800	26.7	173	5.8	819	17.8
*Sphingomonadaceae*	18	0.6	23	0.8	22	0.5
*Xanthomonadaceae*	85	2.8	27	0.9	165	3.6
*Burkholderiaceae*	31	1.0	4	0.1	58	1.3
*Lachnospiraceae*	100	3.3	101	3.4	151	3.3
***Clostridiales Incert. Sed.XI***	145	4.8	11	0.4	126	2.7
***Peptoniphilaceae***	131	4.4	16	0.5	106	2.3
*Pseudomonadaceae*	9	0.3	17	0.6	23	0.5
***Porphyromonadaceae***	76	2.5	52	1.7	94	2.0
*Desulfuromonadales* unclass*.*	29	1.0	16	0.5	48	1.0
***Ruminococcaceae***	82	2.7	30	1.0	21	0.5
***Prevotellaceae***	79	2.6	7	0.2	84	1.8
*Micrococcaceae*	5	0.2	23	0.8	67	1.5
*Alcaligenaceae*	30	1.0	10	0.3	40	0.9
***Clostridiales*unclass.**	61	2.0	7	0.2	60	1.3
*Bacteroidaceae*	43	1.4	29	1.0	32	0.7
*Moraxellaceae*	10	0.3	7	0.2	16	0.3
***Bacteroidales*unclass.**	43	1.4	19	0.6	21	0.5
***Veillonellaceae***	25	0.8	4	0.1	36	0.8

^a^ Families commonly found in VR samples are highlighted in Bold. These were defined as members of the top 25 families found in VR samples (calculated by total reads, see Fig. 4) which were found in 90% of the VR samples examined

**Fig. 8:**
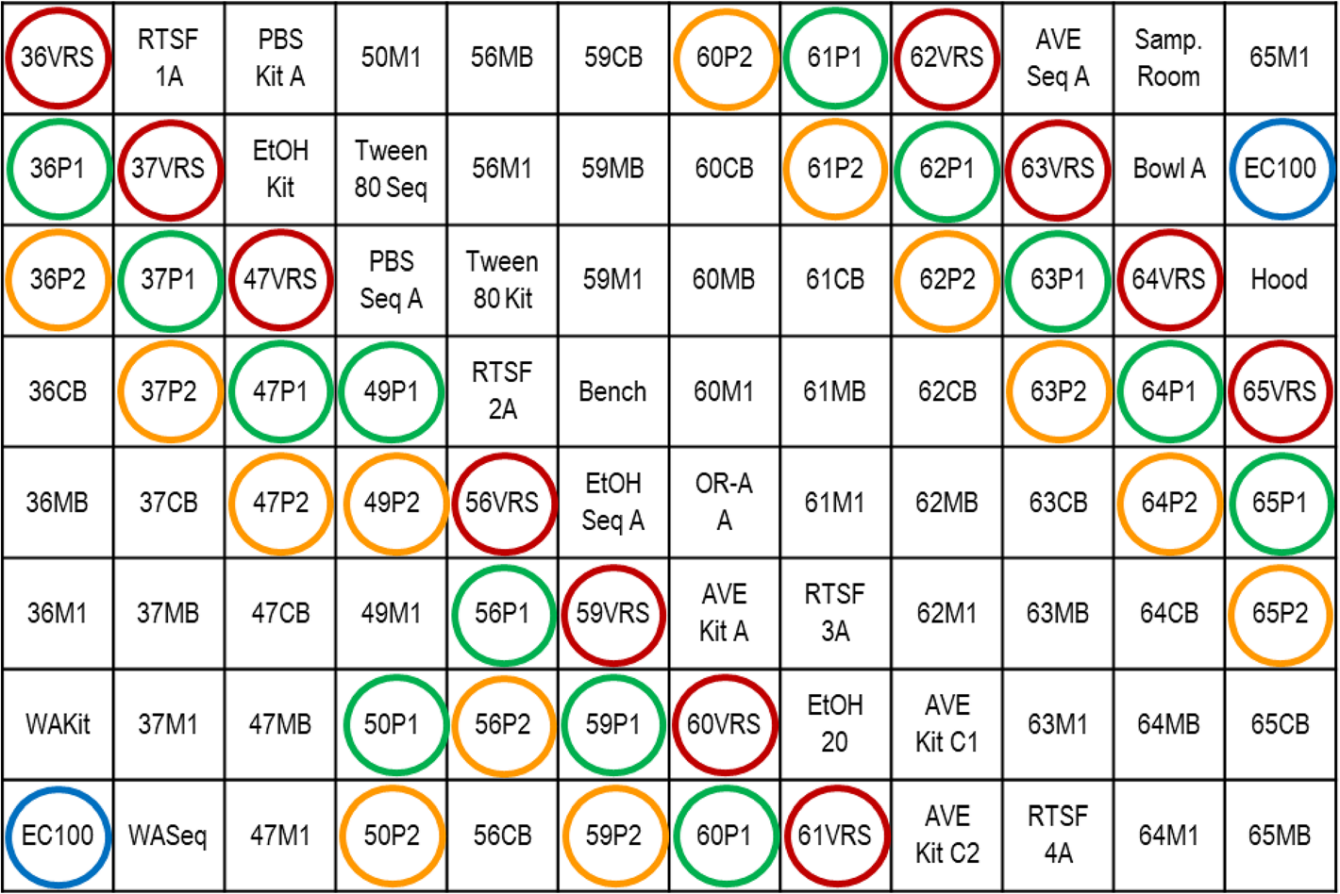
Layout of the samples in one of the 96 well plates submitted for sequencing. The location of P1 samples (circled in GREEN), VR samples (circled in RED), P2 samples (circled in ORANGE), and positive control wells containing *E. coli* (circled in BLUE) are shown

While there were clearly OTUs common to both blanks and samples, such as Enterobacteriaceae, Sphingomonadaceae, Xanthomonadaceae, Burkholderiaceae, and Lachnospiraceae (Table 2), likely due to kit or reagent contamination or “kitome” [12, 21], these did not account for VR OTUs found in P1 samples preferably over P2 samples. To search for an alternative explanation for these results, we examined the layout of samples in the 96 well plates submitted for sequencing (Fig. 8), and we found that P1 samples were commonly placed adjacent to the cognate VR sample, while P2 samples were not. In addition, we found that blank controls placed adjacent to positive control wells containing *E. coli* were frequently also positive for *E. coli*, while blank wells not adjacent to *E. coli* controls were not. Similarly, some blank controls placed adjacent to VR wells were positive for VR organisms. These data suggested that the VR organisms found in P1 samples could be due to contamination, or “splashome”, that occurred during either preparation of the plates or sequencing.

### Repeat sequencing of placental samples

To avoid this putative “splashome”, we repeated the sequencing of placental samples and blanks in a layout where none of these samples was adjacent to a high biomass VR sample or positive control.

In this experiment, placental samples had minimal reads and could not be distinguished from blank controls, either kit controls or sequencing controls. The average reads for P1 samples = 17; for P2 samples = 17; and for blanks = 24. A VR sample run at the same time but placed in a corner of the plate at least 4 wells distant from any placental sample or blank control, yielded 187,777 reads, indicating a successful sequencing run, and very closely matched the results for the same VR sample from the original sequencing run.

The top 20 families found in the placental and blank samples from this repeat sequencing experiment are shown in Table 3. Most of these families are recognized as common contaminants of DNA extraction kits and sequencing processes [12, 20, 21].

**Table 3:** Distribution of bacterial families in resequenced placental samples and blank controls

Family	Placenta (***n*** = 114)	Ave Placenta	Blanks (***n*** = 67)	Ave Blanks
*Sphingomonadaceae*	271	2.4	255	3.8
*Acetobacteraceae*	262	2.3	252	3.8
*Burkholderiales* unclass.	348	3.1	93	1.4
*Burkholderiaceae*	123	1.1	164	2.4
*Xanthomonadaceae*	120	1.1	165	2.5
*Pseudomonadaceae*	153	1.3	41	0.6
*Sphingobacteriaceae*	85	0.7	63	0.9
*Oxalobacteraceae*	106	0.9	33	0.5
*Microbacteriaceae*	69	0.6	60	0.9
*Desulfuromonadales* unclass.	83	0.7	40	0.6
*Staphylococcaceae*	74	0.6	28	0.4
*Alcaligenaceae*	43	0.4	33	0.5
*Moraxellaceae*	43	0.4	28	0.4
*Micrococcaceae*	25	0.2	44	0.7
*Ruminococcaceae*	58	0.5	0	0.0
*Lactobacillaceae*	4	0.0	50	0.7
*Actinomycetales* unclass.	6	0.1	43	0.6
*Shewanellaceae*	21	0.2	17	0.3
*Rhizobiaceae*	36	0.3	0	0.0
*Bacillales unclass.*	25	0.2	10	0.1

## Discussion

The original goal of this study was to determine if a unique placental microbiome exists in pregnant women with gestational diabetes. In our case-control study, placental samples obtained at the time of planned cesarean section from normal weight, obese and gestational diabetic mothers were examined with a view to finding a possible macrosomia-associated placental microbiome in GDM mothers akin to the obesity-associated gut microbiome [9, 10, 27].

In our pilot study, analysis of placental specimens by qPCR indicated the placental samples had higher copy numbers of bacterial 16S rRNA genes than blanks, maternal and cord blood. However, analysis of the microbiome in these samples identified several bacteria, including *Ralstonia, Acinetobacter, Caulobacter, Methylobacterium*, and *Sphingomonas* known to be contaminants in negative controls in previous studies of low-bacterial biomass tissues [13, 18, 21, 22, 28–30], were present in most placental samples as well as in negative controls. In contrast to Aagaard et al. [3], who reported that the placental microbiome was most similar to the oral microbiome, we saw lower levels of Actinobacteria, Bacteroidetes, and Tenericutes. We did identify organisms of possible oral origin, such as *Porphyromonadaceae, Prevotellaceae* and *Streptococcaceae*, but these were minor components of the placental findings in our study. We also noted that organisms that were common in VR samples such as *Lactobacillus, Prevotella*, *Peptoniphilus*, *Ruminococcus*, and other Clostridiales were seen in both placental specimens and blank controls. While there is a chance of ascending spread of these organisms from the vagino-rectal area to the uterine cavity and placenta through intact fetal membranes [31], this mechanism does not explain the detection of these organisms in blank controls. Thus, although the pilot study data indicated that placental samples contained statistically higher bacterial biomass than blank controls, we were unable to clearly differentiate placental samples from blanks based on data obtained in our pilot study. We therefore expanded the study to include 10 subjects in each group, to confirm or reject the presence of a placental microbiome and to determine the possible origin of the placental microbiome if it proved to be present.

In the expanded study, to reduce the level of contamination, we utilized an ultraclean DNA extraction kit. Further, we collected and processed more blanks, including reagents, kit and sequencing reaction controls as well as positive controls on each reaction plate to enable us identify contaminants in reagents and kits [30]. We also included controls in the sequencing reactions and took additional steps to reduce supplies and work area contamination, including wiping down the hood and work bench with bleach as well as 70% ethanol and exposing the hood and supplies to ultra-violet light for at least 1 hour before starting work [32–34]. In this expanded study, real time qPCR analysis showed the VR samples had similar bacterial biomass as in the pilot study while blanks, placental, maternal and cord blood samples had decreased bacterial 16S rRNA gene DNA levels, frequently below the limit of accurate detection. In the pilot study, the DNA samples used for qPCR were certainly affected by kit contamination, but most likely not affected by the splashome effect since we processed DNA extraction on individual samples, not in a 96 well plate. We suspect that increased human cells/DNA in the samples may affect retention of bacterial “kitome” DNA, since the pilot study qPCR data showed placenta > cord blood > maternal blood 16S rRNA gene DNA levels, which parallels the concentration of human cells/DNA in tissues. The kit contamination was greatly reduced in the expanded study, leading to the reduced or undetectable levels seen in all low biomass samples.

In our study, as in the report by *Lauder* et al [12], we were still not able to distinguish a placental bacterial community from blank controls. In their study of placental specimens to investigate the issues of sampling and contamination, Lauder and colleagues analyzed an extensive set of matched experimental and control samples in addition to contamination controls. They also included blank swabs waved in the laboratory air, unused sterile swabs, and included oral and vaginal swabs from the same subjects for comparison. Furthermore, they compared two methods of DNA purification to evaluate for the presence of contaminants in the kits. They reported that placental and negative control samples had very low and indistinguishable bacterial DNA levels by qPCR analysis. In addition, when they compared bacterial lineages in each placental specimen to contamination samples, the results diverged markedly depending on the kit used for DNA purification. Placental samples and negative controls had the same microbial profile depending on the kit used for purification, suggesting that the bacterial profile in the placental samples originated from contaminants in the DNA purification kits.

In addition to contaminants that could be related to reagents and kits, and similar to our finding in the pilot study, we noted that bacteria commonly seen in VR samples were also present in some placental samples and blanks. We thus undertook a careful analysis of the positions of specimens on the sequencing plates and noted that specimen position influenced the results. We found that placement of placental specimens or blanks next to VR or positive controls resulted in detection of microbes from VR or the positive control in the placental specimens or blanks from an apparent ‘splash’ or ‘cross-contamination’ from the high biomass samples. When we repeated the sequencing experiments separating high biomass samples from blanks and placental samples by a distance of at least 4 wells, the occurrence of this “splashome” was essentially eliminated. We reported this observation earlier in a poster presented at the annual conference of the Pediatric Academic Societies (PAS) in 2019 [26]. Our data therefore agrees with the recent report by *Minich* et al [35] of the confounding role of cross-contamination in microbiome studies of low bacterial biomass tissues. Separating high biomass samples from low biomass samples and blanks in microbiome studies that include low and high bacterial biomass tissues or materials represents an easy and inexpensive way of avoiding spurious reports from such studies. Alternatively, it has been suggested that low biomass and high biomass specimens be run on separate sequencing plates to avoid cross-contamination [29].

The problem of reagent and kit contamination in microbiome studies of low bacterial biomass tissues is increasingly recognized and appreciated. Several procedures have been suggested and tried to minimize and control bacterial DNA contamination in such studies. These include: attention to sampling procedures (including gowning and wearing masks to cover operators’ skin and respiratory tract respectively), attention to work environment (wiping down bench and hood with bleach, use of laminar flow hood) [29, 32–34, 36], sample processing (microbial DNA extraction and PCR amplification) [36] and inclusion of negative (blank) and positive controls [12, 21, 30, 32]. Beyond sample collection and sample processing, several bioinformatic techniques have been developed to detect and subtract contaminants in studies of low bacterial biomass tissues but these all have their limitations and little is known so far of the relative effectiveness of the different techniques [37–40]. While it is almost impossible to eliminate reagent, equipment and environmental contamination in microbiome studies, paying attention to sample layout on the sequencing plates appears to be an additional simple and inexpensive step to prevent contamination during the sequencing stage.

Detection of bacteria in the placenta by culture methods in the absence of clinical infection in uncomplicated term pregnancies and delivery was reported more than three decades ago. *Kovalovszky* et al [41] cultured aerobic bacteria from 4% of placentas with no histologic evidence of chorioamnionitis and the newborns and their mothers had no evidence of infection. Subsequently, morphologic studies of human placentas have demonstrated few intracellular bacteria in extravillous trophoblasts of the basal plate in term and preterm deliveries [42, 43]. Also, molecular methods have detected a low abundance microbial community by sequencing of the bacterial 16S rRNA gene [3, 23–25]. However, it is unclear if presence of intracellular bacteria in the basal plate or the detection of low-level bacterial DNA in placental parenchyma constitute evidence for a resident placental bacterial community.

On the other hand, many recent studies with adequate positive and negative controls, utilizing culture, immunohistochemical, molecular and electron microscopic methods, could not identify a placental microbiome [13–15]. *Kuperman* et al [14] in a comprehensive study utilizing bacterial cultures, gram stain, immunohistochemistry, scanning electron microscopy, 16S rRNA gene PCR amplification and TaqMan RT-qPCR only found evidence of a small number of bacterial cells by immunohistochemistry only. The small number of bacteria, which were of questionable viability, was below the limit of detection by the other methods employed and could have represented contaminants acquired during processing. The authors concluded that such small numbers did not provide evidence for true colonization. Similarly, *Theis* et al [13] performed a cross-sectional study of placentas from 29 women delivered at term by cesarean section before onset of labor to evaluate for the presence of a placenta microbiome. They utilized multiple modalities including bacterial culture, quantitative real-time polymerase chain reaction, 16S rRNA gene sequencing and metagenomic surveys. They could not detect consistent differences in the composition or structure of bacterial profiles between placental samples and background technical controls. Twenty-eight of the 29 specimens had negative bacterial culture; the organism retrieved by culture from one specimen was likely a contaminant as corresponding 16S rRNA genes were not detected in the same sample. This study was therefore negative for a resident placental microbiome. In another study, *de Goffau* et al [15] examined placentas from women with pregnancies complicated by pre-eclampsia, preterm delivery or delivery of small for gestational age infants to determine if these complications are associated with the presence of bacterial DNA in the placenta. The authors reported the bacterial biomass obtained from placentas was extremely small and originated mostly from contamination of laboratory reagents and equipment. They found strong evidence for the presence of only *Streptococcus agalactiae* in the placenta before the onset of labor and concluded that bacterial placental infection is not a major cause of placentally related complications of pregnancy, and that the human placenta does not harbor a resident microbiome*.* Similarly, *Leiby* et al [44] could not detect a placental microbiome in placental samples from term and preterm deliveries. Using 16S rRNA gene sequencing and qPCR, they found no significant difference between absolute levels of bacterial DNA in placental samples and negative controls. Furthermore, analysis of bacterial DNA using 16 s rRNA marker gene sequencing or shotgun metagenomic sequencing did not yield a placental microbiome distinguishable from negative controls [44]. However, in a recent report, *Seferovic* et al [45] evaluated placentas from 53 subjects composed of term, preterm, labored and unlabored cesarean deliveries and one placenta from a case of clinical and histologic chorioamnionitis. The preterm cohort included cases of spontaneous preterm deliveries with or without preterm prolonged rupture of fetal membranes as well as medically indicated preterm deliveries. The group reported visualization and localization of low abundance microbes by in situ hybridization probes in placentas in the absence of clinical or histologic chorioamnionitis. They also reported observing 16S rRNA gene signals in 13 of 16 spontaneous preterm placentas that were taxonomically distinct from negative or contamination controls, thereby reconfirming the group’s previous report of a low-abundance microbial community in the placenta [3].

In our study, the placental microbial community in the three groups of mothers did not segregate by study group, and we were unable to distinguish a microbial community in the placentas that differed from contaminants in the blank controls even with the use of an ‘ultra-clean’ DNA extraction kit. The VR bacterial community also did not segregate by study group but by the specific OTU of *Lactobacillus* that was predominant in each sample. We were unable to determine the impact of gestational diabetes on the gut or vaginal microbiota separately due to our sample collection method; we designed the study to mimic as closely as possible the practice in routine obstetric care whereby vaginal-rectal swabs are obtained for Group B *Streptococcus* screening. We plan to obtain separate vaginal and rectal swabs in our future studies to determine the influence of GDM on gut and vaginal microbiota.

A strength of our study is that, in contrast to many studies that reported a placental microbiome [3, 5, 6, 23–25], we enrolled into our study a homogenous population of healthy pregnant women admitted for scheduled cesarean section delivery at term before onset of labor. The subjects had no evidence of infection and no antibiotic use in the months preceding admission. This is important because a significant proportion of spontaneous preterm deliveries are associated with preterm premature rupture of fetal membranes, intrauterine infection and or inflammation [46–48]; furthermore, vaginal delivery increases the chances of placental contamination with recto-vaginal flora.

Another strength of our study is our use of a wide range of positive and negative (blank) controls, in contrast to several studies that reported the presence of a placental microbiome [3, 5, 6, 23, 49]. This is significant because contamination of molecular biology-grade water [50, 51], PCR reagents [18, 52], DNA extraction kits [19, 21], and laboratory equipment [20] confound results and interpretation of data from low bacterial biomass samples. V*an der Horst* et al [20] found a large proportion of two non-oral bacteria, *Enterococcus* and *Exiguobacterium*, in a study of subjects with dental implants, using paper points for sampling. The two non-oral taxa were traced to paper points in a subsequent analysis when two sterile unused paper points were included as blank controls. Failure to control for the presence of contaminants in reagents and equipment is likely to adversely affect the results and interpretation of data from low bacterial biomass samples.

A weakness of our study is the small cohort size of placentas per group, which limited the statistical power of the study. Our original plan was to enroll 30 patients per group, but we ceased enrolling patients once it became clear that there was no placental microbiome distinct from the “kitome” or “splashome” detectable by our methods. Further, obtaining VR specimens instead of separate vaginal and rectal swabs precluded us from noting any effect of GDM or obesity on vaginal or rectal microbiota. However, this was not the primary objective of our study.

## Conclusion

We sought to determine if there exists a unique placental microbiome in pregnant women with gestational diabetes. However, analysis of placental samples obtained at the time of planned cesarean section deliveries revealed the presence of bacteria no different from those in blanks and technical controls. The finding of any significant reads in placental samples was related to either contamination of kit reagents with bacterial DNA (“kitome”), which was reduced but not completely eliminated by use of an ultraclean DNA extraction kit, or the proximity of test samples to high biomass vagino-rectal swab samples on DNA sequencing plates (“splashome”). We determined that having a minimum of 4 wells between high biomass samples and controls and low biomass study samples reduced this well-to-well contamination. Once kitome and splashome contamination was removed, we were unable to confirm the presence of a unique placental microbiome.

## Methods

### Study design and human subjects’ enrollment

We screened the electronic medical records of potential study participants at Sparrow Hospital, Lansing, Michigan to determine eligibility for enrollment into the study. This was a case-control study design to characterize and compare the placental microbiomes of term, gestational diabetic mothers with normal weight and obese mothers. We approached eligible mothers admitted for scheduled cesarean section delivery at term gestation (37^0/7^ to 41^6/7^ weeks) for consent and enrollment into the study. The Michigan State University Institutional Review Board approved the study (IRB# 15-754 M). The study subjects were women with gestational diabetes diagnosed by a three-hour oral glucose tolerance test done after an abnormal screening examination between 24 and 28 weeks’ gestation utilizing criteria described in the relevant American College of Obstetricians and Gynecologists (ACOG) Practice Bulletin [53]. Normal weight (BMI 18.5–24.9) and obese (BMI > 30) mothers also admitted for scheduled cesarean section delivery at term gestation served as controls. All subjects enrolled in the study gave written informed consent to participate in the study. Candidates were excluded from the study if they had any of the following: gestational age < 37 ^0/7^ weeks or > 41 ^6/7^ weeks, late prenatal care or inaccurate gestational dating, onset of labor, rupture of membranes, antibiotic treatment in the third trimester, incomplete medical records including gestational diabetes testing, pregestational diabetes, or pre-pregnancy body mass index (BMI) > 25 but < 30 (overweight but not obese mothers). The obstetric care providers determined the gestational age using the criteria outlined by ACOG [54]. Also, all the subjects were screened for group B *Streptococcus* colonization by their obstetric care providers between 35 and 37 weeks gestation as recommended by the relevant ACOG Committee opinion [55].

For the pilot study, 17 patients (6 GDM, 5 normal BMI and 6 obese) were enrolled. For 1 obese patient, only duplicate placental samples were processed, with no VR, CB, or MB; for a second obese patient, triplicate placental samples were processed in addition to VR, CB, and MB. Therefore, 16 VR, CB, and MB samples and 35 placental samples were analyzed in the pilot study.

For the expanded study, 30 patients (10 in each group) were enrolled. Characteristics of the mothers and infants are shown in Table 1. Only placental samples were available for two of the GDM patients. Therefore, 28 VR, CB, and MB and 60 placental samples were analyzed in the expanded study.

### Maternal and neonatal chart review and data collection

We reviewed each participant’s electronic medical record and extracted demographic data including age, height, pre-pregnancy and 3rd trimester weight, pregnancy weight gain and calculated the pre-pregnancy and 3rd trimester BMI (Weight in Kg)/(Height in M)^2^. We also obtained data from her pre-pregnancy medical history, past obstetric history and history of current pregnancy from her medical records. Following delivery, we obtained delivery data from the mother’s chart and neonatal information from the baby’s chart.

### Specimen collection

Specimens collected for the study included placental parenchyma, maternal vaginal-rectal swabs (VR), maternal blood and cord blood. Placental specimens were obtained under sterile conditions by trained investigators using modifications of the Peribank Manual of Procedures [56] and the method described for placental handling and sampling in the extremely low gestational age newborn (ELGAN) cohort studies [57]. A member of the research team attended the cesarean section delivery to receive the placenta, which was placed in a sterile container with the fetal side up. The placenta was transported to an adjacent clean room for sampling. The investigators obtaining the samples wore masks and sterile gloves. Using sterile tweezers, forceps and scissors, the amnion was gently and entirely pulled away from the chorion. Using a new set of forceps and scissors, the chorion and underlying trophoblast tissue was gently grasped with forceps and a piece of placental tissue was excised with scissors while carefully excluding the maternal surface of the placenta. Finally, a piece of placental parenchyma excluding the chorionic plate was obtained from the block of excised placental tissue. From each placenta, six blocks of parenchymal tissue, each about 1 cm^3^, were excised 3 to 4 cm from cord insertion and placed in 5 ml sterile Cryogenic Storage Vials (Research Products International, Mount Prospect, IL) on dry ice. Cryovials containing placental samples were transported to the laboratory, on dry ice and stored at − 80 °C until analyzed.

The vaginal rectal swabs (Copan Transystem™ 139C, Copan Diagnostics, Murrieta CA) collected during preparation for cesarean section delivery (at the time of bladder catheter placement) were placed in ice-cold 80% ethanol immediately after collection and stored at − 20 °C until analyzed. Maternal blood was drawn at the time of IV-line placement in preparation for cesarean section delivery, while cord blood samples were obtained from the umbilical vein after delivery of the placenta, both using BD Vacutainers #367884, coated with lithium heparin (BD, Franklin Lakes NJ). The blood samples were placed on dry ice immediately after collection, transported to the laboratory on dry ice and stored at − 80 °C until analyzed.

### Specimen processing

#### Placental samples

All placental samples were processed in a laminar flow hood, which had been decontaminated with 10% bleach followed by 70% ethanol and exposure to the germicidal ultraviolet (UV) lights for 1 h prior to use. Each placental sample was thawed and placed in a sterile acid-washed glass petri dish and then finely minced using a pair of sterile scalpels. Duplicate samples of each patient placenta were processed, with the exception that triplicate samples were processed for 1 of the pilot study patients for a total of 35 placental samples for the pilot study. Approximately 200 mg of sample was used for each DNA extraction.

Vaginal rectal swabs in 80% ethanol were vortexed vigorously to remove bacteria from the swab, and the swab discarded. The supernatant was placed into sterile acid washed Corex tubes and centrifuged for 30 min at 12,000 rpm in an SS-34 rotor in a refrigerated Sorvall centrifuge to pellet bacteria. The pellet was suspended in 400 μl Molecular Grade PBS (phosphate buffered saline), and the full sample processed for DNA extraction [58].

In the pilot study, blood specimens were thawed and 6 ml of 0.17 M ammonium chloride was added to 1.5 ml of blood sample and incubated for 20 min at 37 °C to lyse the red blood cells (RBCs). The samples were centrifuged for 10 min at 4000 rpm at 4 °C, the supernatant discarded, and the pellet suspended in 400 μl molecular grade PBS and added directly to the PowerSoil bead tubes for DNA extraction. In the expanded study, blood specimens were thawed and 4 ml was mixed with 10 ml of cold Red Blood Cell (RBC) lysing solution (0.8% NH_4_CL, 0.08% NaHCO_3,_ 0.04% disodium EDTA in Molecular Grade water, filter sterilized), vortexed vigorously and incubated for 30 min at 37 °C to lyse the RBCs prior to DNA extraction. The samples were centrifuged for 10 min at 4000 rpm at 4 °C, the supernatant discarded, and the pellet suspended in 400 μl molecular grade PBS, and the full sample processed for DNA extraction in the QIAamp kit.

### DNA extraction

Two different DNA extraction kits were used in this study.

For the pilot study, we used the Mo-Bio PowerSoil DNA Isolation Kit (Mo-Bio Laboratories), which has been routinely used in Human Microbiome Project studies; all kits were from the same lot number. In addition to the placental, VR, and blood samples, multiple controls were processed through the MoBio Kit to evaluate kit and reagent contamination. These included MoBio kit reagents, ethanol, ammonium chloride, sterile swabs (identical to those used for VR samples) passed through the air in the operating room and the sampling room, and blank sterile swabs. These negative controls (a total of 13), while analyzed independently to assess contamination due to reagents, kit, sequencing, and sampling, were grouped together as “blanks” in the analyses presented.

For the expanded study, we used QIAamp UCP Pathogen Mini kits (Qiagen), combined with Pathogen Lysis Tubes S (Qiagen), following the manufacturer’s instructions. Again, all kits of each type were from the same lot number, as were each of the reagents used. Multiple reagent, DNA extraction and sequencing controls were run. These included sterile ultrapure water, PBS, ethanol, RBC lysing solution, and the AVE elution buffer from the Qiagen kits (a sample from each of the kits used), both alone (reagent controls) and processed through the kits (extraction or kit controls). In addition, swabs were used to sample the bowl used to collect and transport the placenta, the air of the operating room and the sampling room, and the hood. These negative controls (a total of 53), while analyzed independently to assess contamination due to reagents, kit, sequencing, and sampling, were grouped together as “blanks” in the analyses presented. Samples (400 μl from each blood or VR and 200 mg of placental tissue) were first processed through the Pathogen Lysis Tube S with vortexing for 10 min at maximum speed. Proteinase K (40 μl) was added to each sample. Samples were then incubated at 56 °C for 10 min and then processed through the QIAamp Pathogen Mini Kit. DNA concentration was measured using a Qubit.

Since the Qiagen QiaAMP UCP Pathogen Mini Kit had not been validated for use with tissue at the initiation of this project, we performed multiple test runs with placental tissues and modified procedures to ensure that the extracted DNA profiles we achieved, as visualized on agarose gels, matched the profiles we had previously achieved with the MoBio Power Soil kit.

### 16S rRNA gene sequencing and analysis

For the pilot study, samples were processed for sequencing of the V3-V4 region of the bacterial 16S rRNA gene using an Illumina MiSeq platform with paired 2 X 250 bp reads, at SeqMatic (Fremont CA). Illumina reads (about 10 million) with an average read length of 455 ± 48 bp were processed with QIIME2 [59]. Deblur was used after quality filtering and demultiplexing for quality control as well as feature table construction. Phylogenetic analysis was also carried out in QIIME2 with classifier trained for our dataset with V3-V4 primers. Statistical analyses were performed with PAST3 (Paleontological Statistics Software Package For Education and Data Analysis) [60]. The Metadata and the code used for the analysis using QIIME2 are available at https://figshare.com/s/dcff5b4c7e8a54dfdd6e.

For the expanded study, samples were processed for sequencing of the V4 region of the bacterial 16S rRNA gene from the community DNA using an Illumina MiSeq v2 platform with paired 2 X 250 bp reads, at the Michigan State University Research Technology Support Facility (RTSF). Sequencing controls consisting of RT-grade water were added to each reaction plate. Libraries targeting the V4 hypervariable region of the 16S rRNA gene were prepared using dual indexed Illumina compatible primers 515f/806r following a standard protocol [61]. In a preliminary test experiment, amplicon libraries were prepared using 25, 30 or 35 cycles of amplification. Further, completed amplicon libraries were either 1) bulk normalized using Invitrogen SequalPrep DNA Normalization plates and pooled, and the pool cleaned using AmpureXP magnetic beads; or 2) not normalized. Further, the effect of mixing samples by pipetting up and down on splash of samples into adjacent wells was tested. Based on the results of these test experiments, amplicon libraries for the complete set of samples and controls were constructed using 30 cycles of amplification and were not normalized prior to sequencing and were not mixed by pipetting. Base calling was done by Illumina Real Time Analysis (RTA) v1.18.54 and output of RTA was demultiplexed and converted to FastQ format with Illumina Bcl2fastq v2.19.1.

The open-source software program Mothur v.1.39.5 [62] was used for amplicon analysis. Raw sequencing data were processed according to the Mothur standard operating procedure [61]. Alignment of the sequences was done using the Mothur-formatted version 123 of Silva 16S rRNA gene database [63]. After sequences were classified, all sequences classified as Chloroplast, Mitochondria, unknown, Archaea, or Eukaryota were removed from the data set. A Mothur formatted version of the Ribosomal Database Project (RDP) training set version 16 and Uchime were used based on Mothur protocol to do preclustering of the sequences and removal of chimeric sequences. A cutoff of ≥97% sequence identity was used to classify sequences into Operational Taxonomic Units (OTUs). Singleton and doubleton reads were removed before the final analysis. Statistical analyses were performed with PAST3 (Paleontological Statistics Software Package for Education and Data Analysis [60].

The full data set analyzed is available as an NIH BioProject at https://www.ncbi.nlm.nih.gov/sra/PRJNA577959. The Mothur code used and raw sequence data (proportions) are available at https://figshare.com/articles/Full_study/9992522 .

### 16S rRNA gene qPCR

To quantify bacterial loads in the communities, quantitative PCR (qPCR) was performed using 16S rRNA gene universal primers 357F (5′-CTCCTACGGGAGGCAGCAG-3′) and 519R (536R) (5′-GWATTACCGCGGCKGCTG-3′) [64]. Reactions with SsoAdvanced™ Universal SYBR™Green Supermix (BioRad) were performed in triplicate in a 15 μl reaction, using 1:2 dilutions of DNA template. An initial denaturation at 95 °C for 20 s was followed by 40 cycles of 3 s at 95 °C and 30 s at 55 °C. Boxplots were plotted with SigmaPlot 10.0 and 14.0 (Systat Software, Inc) for the pilot and expanded study respectively. Jitter plot was constructed in RStudio V. 1.0.136 with ggplot2 library. The control plasmid containing a single copy of the 16S rRNA gene was kindly provided by Dr. Fredric Bushman and Jacob Leiby at the University of Pennsylvania [12]. For the expanded study samples were run in quadruplicates as above or using PowerUp™ SYBR™ Green Master mix (Applied Biosystems™). For determining significant differences in groups based on qPCR, ANOVA test followed by Tukey’s test was performed in PAST3 [60].

## Supplementary information

**Additional file 1: Fig. S1.** The VR microbial community from the pilot study samples at the phylum level. Colored bars illustrate the percentage of total reads classified into specific phyla. The last column in each group shows the average for that set of patients. These average columns are repeated on the far right for ease of comparison. Note that VR samples from this pilot study included 1 from each of 16 patients.

**Additional file 2: Fig. S2.** The VR microbial community from the pilot study samples at the family level. The top 20 families (of 41 total identified) found in VR communities are shown. Colored bars illustrate the percentage of total reads classified into specific families. The last column in each group shows the average for that set of patients. These average columns are repeated on the far right for ease of comparison. Families are color coded to match the phyla in Supplemental Fig. 1, e.g., families in the phylum Firmicutes are depicted in shades of blue while families in the Phylum Proteobacteria are depicted in shades of green. Uncl. = unclassified. Note that VR samples from this pilot study included 1 from each of 16 patients.

**Additional file 3: Fig. S3.** The placental microbial community from the pilot study samples at the phylum level. Colored bars illustrate the percentage of total reads classified into specific phyla. The last column in each group shows the average for that set of blank controls or patients. These average columns are repeated on the far right for ease of comparison, and are labeled B = blank, G = GDM, N = normal, and O = obese. Note that placental samples from this pilot study included 2 each from 16 patients and triplicate samples from 1 patient for a total of 35.

**Additional file 4: Fig. S4.** The placental microbial community from the pilot study samples at the family level. The top 25 families (of 41 total identified) are shown. Colored bars illustrate the percentage of total reads classified into specific families. The last column in each group shows the average for that set of patients or blank controls. These average columns are repeated on the far right for ease of comparison, and are labeled B = blank, G = GDM, N = normal, and O = obese. Families are color coded to match the phyla in Fig. 3, e.g., families in the phylum Firmicutes are depicted in shades of blue while families in the Phylum Proteobacteria are depicted in shades of green. Uncl. = unclassified. Note that placental samples from this pilot study included 2 each from 16 patients and triplicate samples from 1 patient for a total of 35.

**Additional file 5: Fig. S5.** The microbial community at the family level in samples from the expanded study. The top 25 families (of 90 total identified) in two separate placental samples (P1 and P2), cord blood (CB), maternal blood (MB) and blanks from GDM, normal BMI, and obese patients, are shown. Each column shows the sum of all samples in that group, i.e., 10 P1, P2, CB and MB samples for each group, except GDM CB and MB which contain only 8 patient samples each. Blanks contain 53 separate samples. Total reads for each set of samples is indicated below each column. Colored bars illustrate the percentage of total reads classified into specific families. Families are color coded to match the phyla in previous figures, e.g., families in the phylum Firmicutes are depicted in shades of blue while families in the Phylum Proteobacteria are depicted in shades of green. Uncl. = unclassified; Inc. Sed. = Incertae Sedis.

## Data Availability

The datasets generated and analyzed during the current study are available in the NCBI SRA accession number PRJNA577959 and in Figshare.

## Abbreviations

GDM: Gestational diabetes mellitus
BMI: Body mass index
NH_4_Cl: Ammonium chloride
EtOH: Ethanol
GTT: Glucose tolerance test
GBS: Group B streptococcus
qPCR: Quantitative polymerase chain reaction
VR: Vagina rectal
OTUs: Operational taxonomic units
ACOG: American College of Obstetricians and Gynecologists
ELGAN: Extremely low gestational age newborn
PBS: Phosphate buffered saline
UV: Ultra violet
RBC: Red blood cell
RTA: Real time analysis
rpm: Revolutions per minute

## References

[1] Consortium THMP (2012). Structure, function and diversity of the healthy human microbiome. Nature.

[2] NIH Human Microbiome Project [https://hmpdacc.org/hmp/]. Accessed Dec 2019.

[3] Aagaard K, Ma J, Antony KM, Ganu R, Petrosino J, Versalovic J (2014). The placenta harbors a unique microbiome. Sci Transl Med.

[4] Radaelli T, Lepercq J, Varastehpour A, Basu S, Catalano PM, Hauguel-De Mouzon S (2009). Differential regulation of genes for fetoplacental lipid pathways in pregnancy with gestational and type 1 diabetes mellitus. Am J Obstet Gynecol.

[5] Bassols J, Serino M, Carreras-Badosa G, Burcelin R, Blasco-Baque V, Lopez-Bermejo A, Fernandez-Real JM (2016). Gestational diabetes is associated with changes in placental microbiota and microbiome. Pediatr Res.

[6] Zheng J, Xiao X, Zhang Q, Mao L, Yu M, Xu J, Wang T (2017). The placental microbiota is altered among subjects with gestational diabetes mellitus: a pilot study. Front Physiol.

[7] Zheng J, Xiao XH, Zhang Q, Mao LL, Yu M, Xu JP, Wang T (2017). Correlation of placental microbiota with fetal macrosomia and clinical characteristics in mothers and newborns. Oncotarget.

[8] Moreno-Indias I, Cardona F, Tinahones FJ, Queipo-Ortuno MI (2014). Impact of the gut microbiota on the development of obesity and type 2 diabetes mellitus. Front Microbiol.

[9] Turnbaugh PJ, Backhed F, Fulton L, Gordon JI (2008). Diet-induced obesity is linked to marked but reversible alterations in the mouse distal gut microbiome. Cell Host Microbe.

[10] Turnbaugh PJ, Hamady M, Yatsunenko T, Cantarel BL, Duncan A, Ley RE, Sogin ML, Jones WJ, Roe BA, Affourtit JP (2009). A core gut microbiome in obese and lean twins. Nature.

[11] Bouter KE, van Raalte DH, Groen AK, Nieuwdorp M (2017). Role of the gut microbiome in the pathogenesis of obesity and obesity-related metabolic dysfunction. Gastroenterology.

[12] Lauder AP, Roche AM, Sherrill-Mix S, Bailey A, Laughlin AL, Bittinger K, Leite R, Elovitz MA, Parry S, Bushman FD (2016). Comparison of placenta samples with contamination controls does not provide evidence for a distinct placenta microbiota. Microbiome.

[13] Theis KR, Romero R, Winters AD, Greenberg JM, Gomez-Lopez N, Alhousseini A, Bieda J, Maymon E, Pacora P, Fettweis JM (2019). Does the human placenta delivered at term have a microbiota? Results of cultivation, quantitative real-time PCR, 16S rRNA gene sequencing, and metagenomics. Am J Obstet Gynecol.

[14] Kuperman AA, Zimmerman A, Hamadia S, Ziv O, Gurevich V, Fichtman B, Gavert N, Straussman R, Rechnitzer H, Barzilay M, et al. Deep microbial analysis of multiple placentas shows no evidence for a placental microbiome. BJOG. 2019. 10.1111/1471-0528.15896. 10.1111/1471-0528.15896 31376240

[15] de Goffau MC, Lager S, Sovio U, Gaccioli F, Cook E, Peacock SJ, Parkhill J, Charnock-Jones DS, Smith GCS (2019). Human placenta has no microbiome but can contain potential pathogens. Nature.

[16] Potgieter M, Bester J, Kell DB, Pretorius E (2015). The dormant blood microbiome in chronic, inflammatory diseases. FEMS Microbiol Rev.

[17] Kliman HJ (2014). Comment on "the placenta harbors a unique microbiome". Sci Transl Med.

[18] Grahn N, Olofsson M, Ellnebo-Svedlund K, Monstein HJ, Jonasson J (2003). Identification of mixed bacterial DNA contamination in broad-range PCR amplification of 16S rDNA V1 and V3 variable regions by pyrosequencing of cloned amplicons. FEMS Microbiol Lett.

[19] Mohammadi T, Reesink HW, Vandenbroucke-Grauls CM, Savelkoul PH (2005). Removal of contaminating DNA from commercial nucleic acid extraction kit reagents. J Microbiol Methods.

[20] van der Horst J, Buijs MJ, Laine ML, Wismeijer D, Loos BG, Crielaard W, Zaura E (2013). Sterile paper points as a bacterial DNA-contamination source in microbiome profiles of clinical samples. J Dent.

[21] Salter SJ, Cox MJ, Turek EM, Calus ST, Cookson WO, Moffatt MF, Turner P, Parkhill J, Loman NJ, Walker AW (2014). Reagent and laboratory contamination can critically impact sequence-based microbiome analyses. BMC Biol.

[22] Glassing A, Dowd SE, Galandiuk S, Davis B, Chiodini RJ (2016). Inherent bacterial DNA contamination of extraction and sequencing reagents may affect interpretation of microbiota in low bacterial biomass samples. Gut Pathog.

[23] Antony KM, Ma J, Mitchell KB, Racusin DA, Versalovic J, Aagaard K (2015). The preterm placental microbiome varies in association with excess maternal gestational weight gain. Am J Obstet Gynecol.

[24] Gomez-Arango LF, Barrett HL, McIntyre HD, Callaway LK, Morrison M, Nitert MD (2017). Contributions of the maternal oral and gut microbiome to placental microbial colonization in overweight and obese pregnant women. Sci Rep.

[25] Parnell LA, Briggs CM, Cao B, Delannoy-Bruno O, Schrieffer AE, Mysorekar IU (2017). Microbial communities in placentas from term normal pregnancy exhibit spatially variable profiles. Sci Rep.

[26] Olomu IN, Pena-Cortes LC, Long R, Singh P, Vyas A, Krichevsky O, Mulks MH (2019). Failure to Detect a Placental Microbiome.

[27] Turnbaugh PJ, Ley RE, Mahowald MA, Magrini V, Mardis ER, Gordon JI (2006). An obesity-associated gut microbiome with increased capacity for energy harvest. Nature.

[28] Laurence M, Hatzis C, Brash DE (2014). Common contaminants in next-generation sequencing that hinder discovery of low-abundance microbes. PLoS One.

[29] Eisenhofer R, Minich JJ, Marotz C, Cooper A, Knight R, Weyrich LS (2019). Contamination in low microbial biomass microbiome studies: issues and recommendations. Trends Microbiol.

[30] Kim D, Hofstaedter CE, Zhao C, Mattei L, Tanes C, Clarke E, Lauder A, Sherrill-Mix S, Chehoud C, Kelsen J (2017). Optimizing methods and dodging pitfalls in microbiome research. Microbiome.

[31] Goncalves LF, Chaiworapongsa T, Romero R (2002). Intrauterine infection and prematurity. Ment Retard Dev Disabil Res Rev.

[32] Corless CE, Guiver M, Borrow R, Edwards-Jones V, Kaczmarski EB, Fox AJ (2000). Contamination and sensitivity issues with a real-time universal 16S rRNA PCR. J Clin Microbiol.

[33] Gefrides LA, Powell MC, Donley MA, Kahn R (2010). UV irradiation and autoclave treatment for elimination of contaminating DNA from laboratory consumables. Forensic Sci Int Genet.

[34] Ohta J, Tanaka A (2018). Elimination of contaminating amplified short tandem repeat products by autoclaving and ultraviolet irradiation. Med Sci Law.

[35] Minich JJ, Sanders JG, Amir A, Humphrey G, Gilbert JA, Knight R. Quantifying and Understanding Well-to-Well Contamination in Microbiome Research. mSystems. 2019;4;e00186–19. 10.1128/mSystems.00186-19. 10.1128/mSystems.00186-19 PMC6593221 31239396

[36] Champlot S, Berthelot C, Pruvost M, Bennett EA, Grange T, Geigl EM. An efficient multistrategy DNA decontamination procedure of PCR reagents for hypersensitive PCR applications. PLoS One. 2010;5(9):e13042. 10.1371/journal.pone.0013042. 10.1371/journal.pone.0013042 PMC2946917 20927390

[37] Kostic AD, Ojesina AI, Pedamallu CS, Jung J, Verhaak RG, Getz G, Meyerson M (2011). PathSeq: software to identify or discover microbes by deep sequencing of human tissue. Nat Biotechnol.

[38] Zhou Q, Su X, Ning K (2014). Assessment of quality control approaches for metagenomic data analysis. Sci Rep.

[39] Minich JJ, Zhu Q, Janssen S, Hendrickson R, Amir A, Vetter R, Hyde J, Doty MM, Stillwell K, Benardini J, et al. KatharoSeq Enables High-Throughput Microbiome Analysis from Low-Biomass Samples. mSystems. 2018;3:e00218–17. 10.1128/mSystems.00218-17. 10.1128/mSystems.00218-17 PMC5864415 29577086

[40] Marsh RL, Nelson MT, Pope CE, Leach AJ, Hoffman LR, Chang AB, Smith-Vaughan HC (2018). How low can we go? The implications of low bacterial load in respiratory microbiota studies. Pneumonia (Nathan).

[41] Kovalovszki L, Villanyi Z, Pataki I, Veszelowvsky I, Nagy ZB (1982). Isolation of aerobic bacteria from the placenta. Acta Paediatr Acad Sci Hung.

[42] Cao B, Mysorekar IU (2014). Intracellular bacteria in placental basal plate localize to extravillous trophoblasts. Placenta.

[43] Stout MJ, Conlon B, Landeau M, Lee I, Bower C, Zhao Q, Roehl KA, Nelson DM, Macones GA, Mysorekar IU (2013). Identification of intracellular bacteria in the basal plate of the human placenta in term and preterm gestations. Am J Obstet Gynecol.

[44] Leiby JS, McCormick K, Sherrill-Mix S, Clarke EL, Kessler LR, Taylor LJ, Hofstaedter CE, Roche AM, Mattei LM, Bittinger K (2018). Lack of detection of a human placenta microbiome in samples from preterm and term deliveries. Microbiome.

[45] Seferovic MD, Pace RM, Carroll M, Belfort B, Major AM, Chu DM, Racusin DA, Castro ECC, Muldrew KL, Versalovic J (2019). Visualization of microbes by 16S in situ hybridization in term and preterm placentas without intraamniotic infection. Am J Obstet Gynecol.

[46] Romero R, Gomez R, Chaiworapongsa T, Conoscenti G, Kim JC, Kim YM (2001). The role of infection in preterm labour and delivery. Paediatr Perinat Epidemiol.

[47] Goldenberg RL, Culhane JF, Iams JD, Romero R (2008). Epidemiology and causes of preterm birth. Lancet.

[48] Boyle AK, Rinaldi SF, Norman JE, Stock SJ (2017). Preterm birth: inflammation, fetal injury and treatment strategies. J Reprod Immunol.

[49] Amarasekara R, Jayasekara RW, Senanayake H, Dissanayake VH (2015). Microbiome of the placenta in pre-eclampsia supports the role of bacteria in the multifactorial cause of pre-eclampsia. J Obstet Gynaecol Res.

[50] McAlister MB, Kulakov LA, O'Hanlon JF, Larkin MJ, Ogden KL (2002). Survival and nutritional requirements of three bacteria isolated from ultrapure water. J Ind Microbiol Biotechnol.

[51] McFeters GA, Broadaway SC, Pyle BH, Egozy Y (1993). Distribution of bacteria within operating laboratory water purification systems. Appl Environ Microbiol.

[52] Newsome T, Li BJ, Zou N, Lo SC (2004). Presence of bacterial phage-like DNA sequences in commercial Taq DNA polymerase reagents. J Clin Microbiol.

[53] Committee on Practice B-O (2018). ACOG Practice Bulletin No. 190: Gestational Diabetes Mellitus. Obstet Gynecol.

[54] Committee on Practice B-O, the American Institute of Ultrasound in M (2016). Practice Bulletin No. 175: Ultrasound in Pregnancy. Obstet Gynecol.

[55] Prevention of Group B Streptococcal Early-Onset Disease in Newborns (2020). ACOG Committee opinion, number 797. Obstet Gynecol.

[56] Antony KM, Hemarajata P, Chen J, Morris J, Cook C, Masalas D, Gedminas M, Brown A, Versalovic J, Aagaard K (2016). Generation and validation of a universal perinatal database and biospecimen repository: PeriBank. J Perinatol.

[57] Onderdonk AB, Delaney ML, DuBois AM, Allred EN, Leviton A (2008). Extremely Low Gestational Age Newborns Study I: Detection of bacteria in placental tissues obtained from extremely low gestational age neonates. Am J Obstet Gynecol.

[58] Pena Cortes LC, LeVeque RM, Funk J, Marsh TL, Mulks MH (2018). Development of the tonsillar microbiome in pigs from newborn through weaning. BMC Microbiol.

[59] Hall M, Beiko RG (1849). 16S rRNA gene analysis with QIIME2. Methods Mol Biol.

[60] Hammer O, Harper DAT, Ryan PD (2001). PAST: paleontological statistics software package for education and data analysis. Paleontologia Electronica.

[61] Kozich JJ, Westcott SL, Baxter NT, Highlander SK, Schloss PD (2013). Development of a dual-index sequencing strategy and curation pipeline for analyzing amplicon sequence data on the MiSeq Illumina sequencing platform. Appl Environ Microbiol.

[62] Schloss PD, Westcott SL, Ryabin T, Hall JR, Hartmann M, Hollister EB, Lesniewski RA, Oakley BB, Parks DH, Robinson CJ (2009). Introducing mothur: open-source, platform-independent, community-supported software for describing and comparing microbial communities. Appl Environ Microbiol.

[63] Quast C, Pruesse E, Yilmaz P, Gerken J, Schweer T, Yarza P, Peplies J, Glockner FO (2013). The SILVA ribosomal RNA gene database project: improved data processing and web-based tools. Nucleic Acids Res.

[64] Turner S, Pryer KM, Miao VP, Palmer JD (1999). Investigating deep phylogenetic relationships among cyanobacteria and plastids by small subunit rRNA sequence analysis. J Eukaryot Microbiol.

