# Supplementary material for: Elimination of “kitome” and “splashome” contamination results in lack of detection of a unique placental microbiome
Source: BMC Microbiol. 2020 Jun 11;20:157. doi: 10.1186/s12866-020-01839-y (PMC7291729; doi:10.1186/s12866-020-01839-y)
Supplement: Supplementary file 1 [file 12866_2020_1839_MOESM1_ESM.docx]

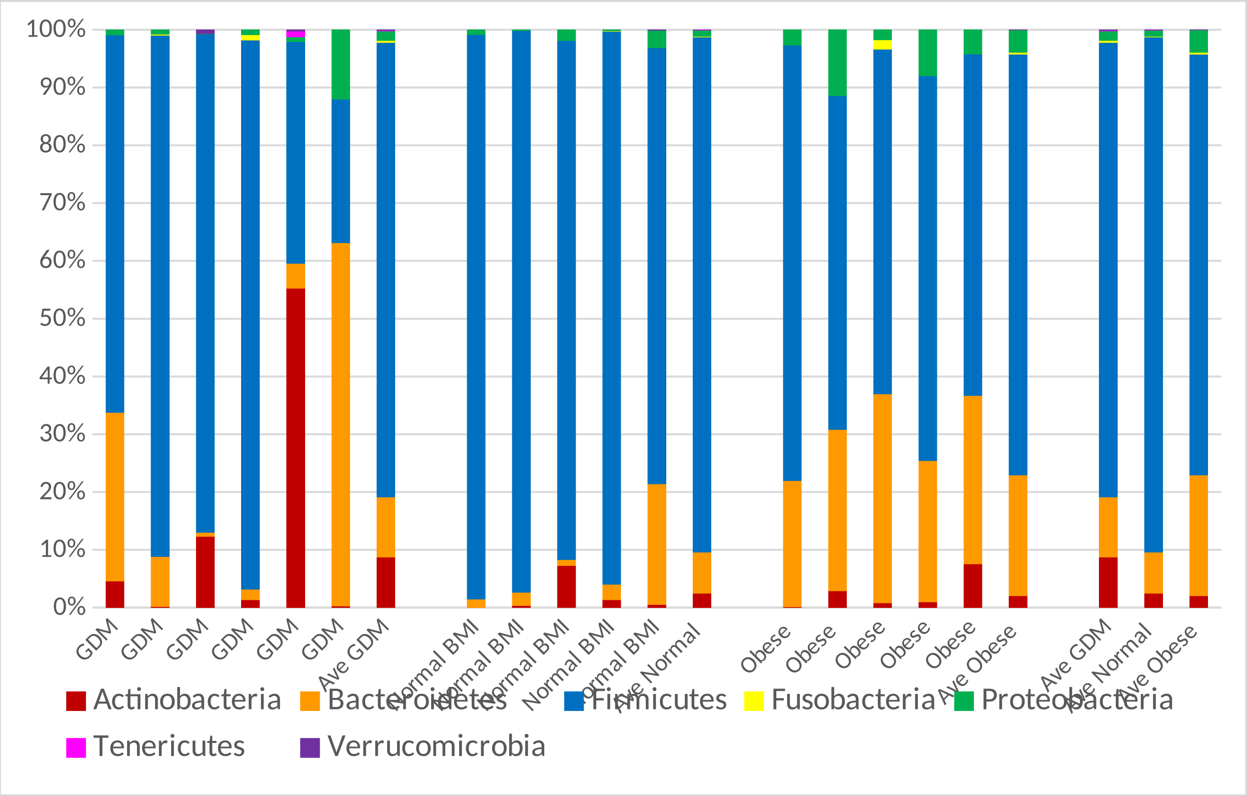

100%
90%
80%
70%
60%
50%
40%
30%
20%
10%
0%
GDM
GDM
GDM
GDM
GDM
GDM
Ave GDM
Normal BMI
Normal BMI
Normal BMI
Normal BMI
Normal BMI
Ave Normal
Obese
Obese
Obese
Obese
Obese
Ave Obese
Ave GDM
Ave Normal
Ave Obese
Actinobacteria
Bacteroidetes
Firmicutes
Fusobacteria
Proteobacteria
Tenericutes
Verrucomicrobia
