# Supplementary material for: Elimination of “kitome” and “splashome” contamination results in lack of detection of a unique placental microbiome
Source: BMC Microbiol. 2020 Jun 11;20:157. doi: 10.1186/s12866-020-01839-y (PMC7291729; doi:10.1186/s12866-020-01839-y)
Supplement: Supplementary file 2 [file 12866_2020_1839_MOESM2_ESM.docx]

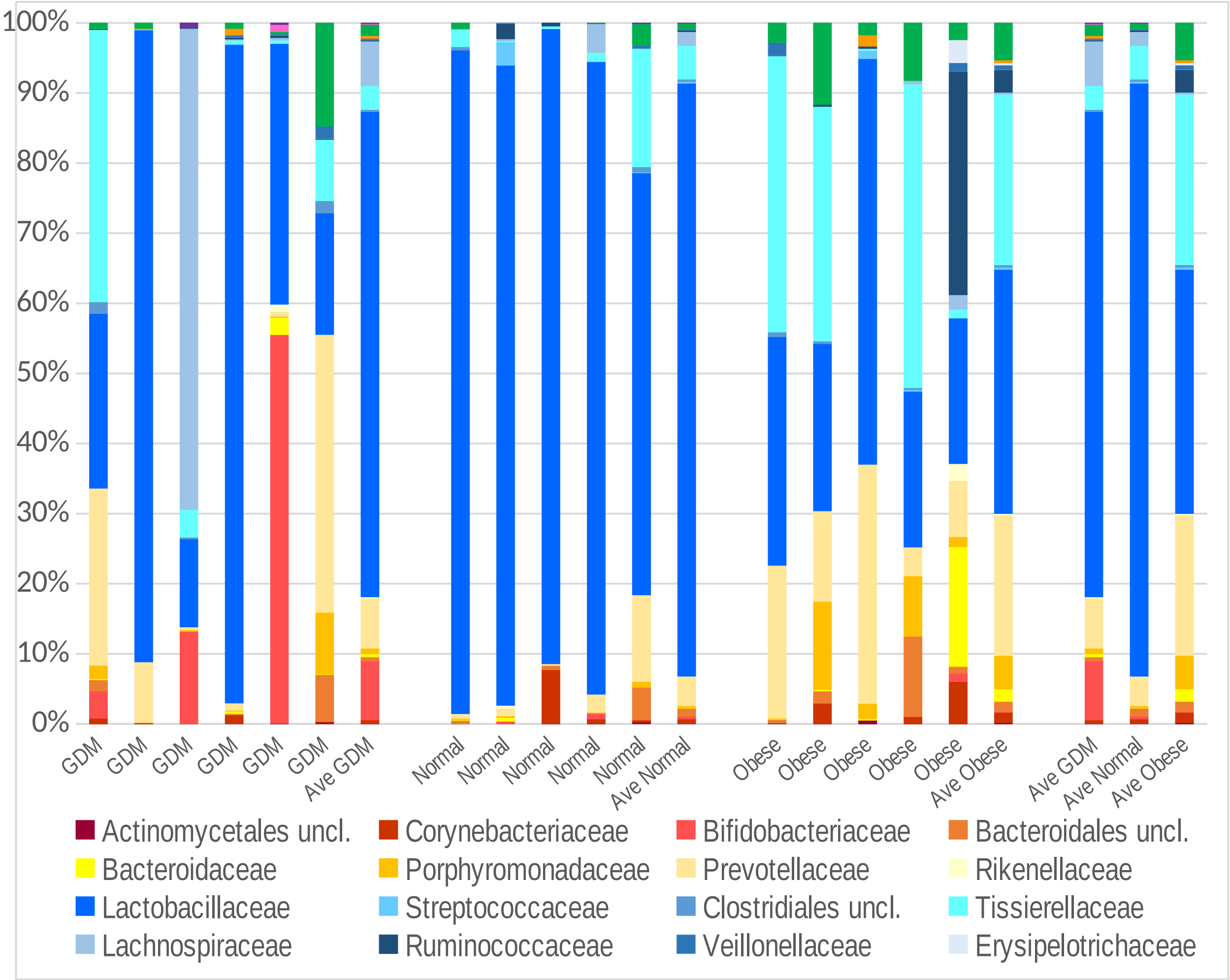
100%
90%
80%
70%
60%
50%
40%
30%
20%
10%
0%
GDM
GDM
GDM
GDM
GDM
GDM
Ave GDM
Normal
Normal
Normal
Normal
Normal
Ave Normal
Obese
Obese
Obese
Obese
Obese
Ave Obese
Ave GDM
Ave Normal
Ave Obese
Actinomycetales uncl.
Corynebacteriaceae
Bifidobacteriaceae
Bacteroidales uncl.
Bacteroidaceae
Porphyromonadaceae
Prevotellaceae
Rikenellaceae
Lactobacillaceae
Streptococcaceae
Clostridiales uncl.
Tissierellaceae
Lachnospiraceae
Ruminococcaceae
Veillonellaceae
Erysipelotrichaceae
