# Supplementary material for: Elimination of “kitome” and “splashome” contamination results in lack of detection of a unique placental microbiome
Source: BMC Microbiol. 2020 Jun 11;20:157. doi: 10.1186/s12866-020-01839-y (PMC7291729; doi:10.1186/s12866-020-01839-y)
Supplement: Supplementary file 3 [file 12866_2020_1839_MOESM3_ESM.docx]

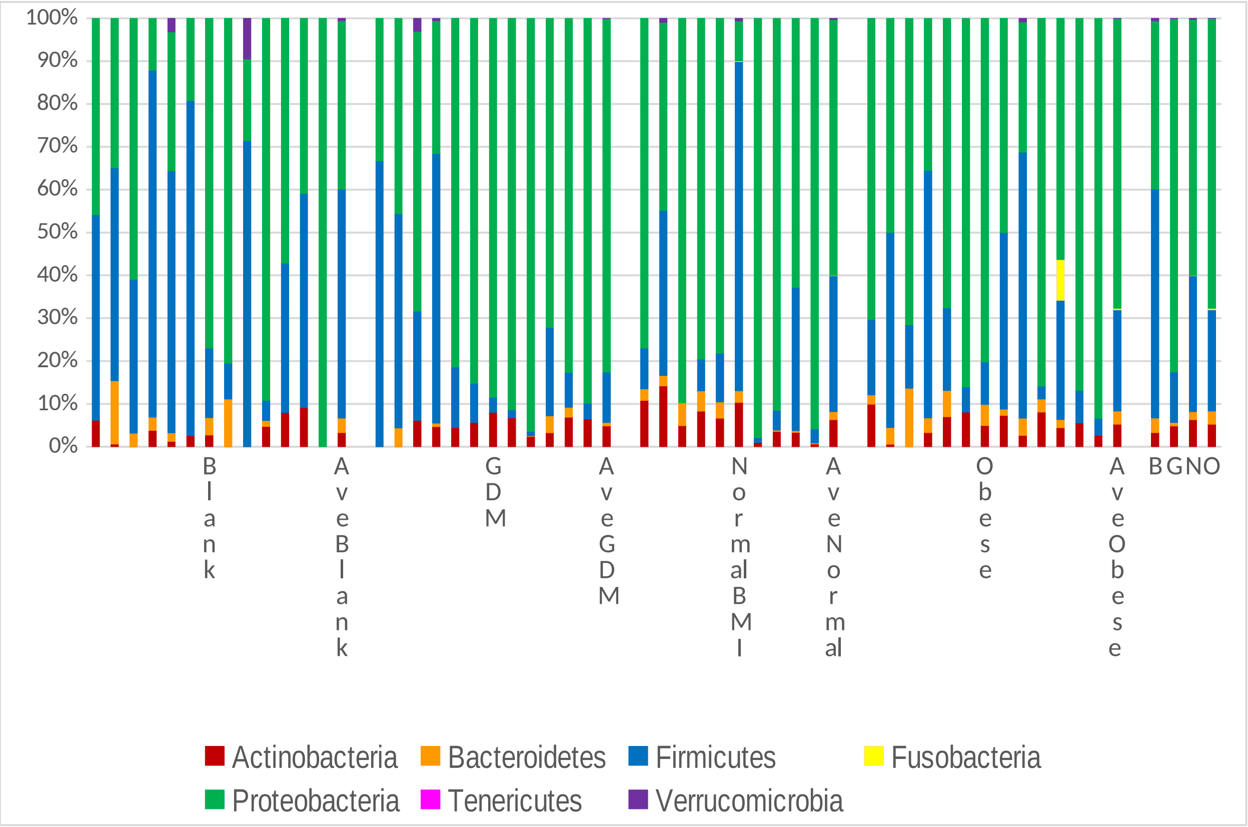

100%
90%
80%
70%
60%
50%
40%
30%
20%
10%
0%
Blank
AveBlank
GDM
AveGDM
NormalBMI
AveNormal
Obese
AveObese
BGNO
Actinobacteria
Bacteroidetes
Firmicutes
Fusobacteria
Proteobacteria
Tenericutes
Verrucomicrobia
