# Supplementary material for: Elimination of “kitome” and “splashome” contamination results in lack of detection of a unique placental microbiome
Source: BMC Microbiol. 2020 Jun 11;20:157. doi: 10.1186/s12866-020-01839-y (PMC7291729; doi:10.1186/s12866-020-01839-y)
Supplement: Supplementary file 4 [file 12866_2020_1839_MOESM4_ESM.docx]

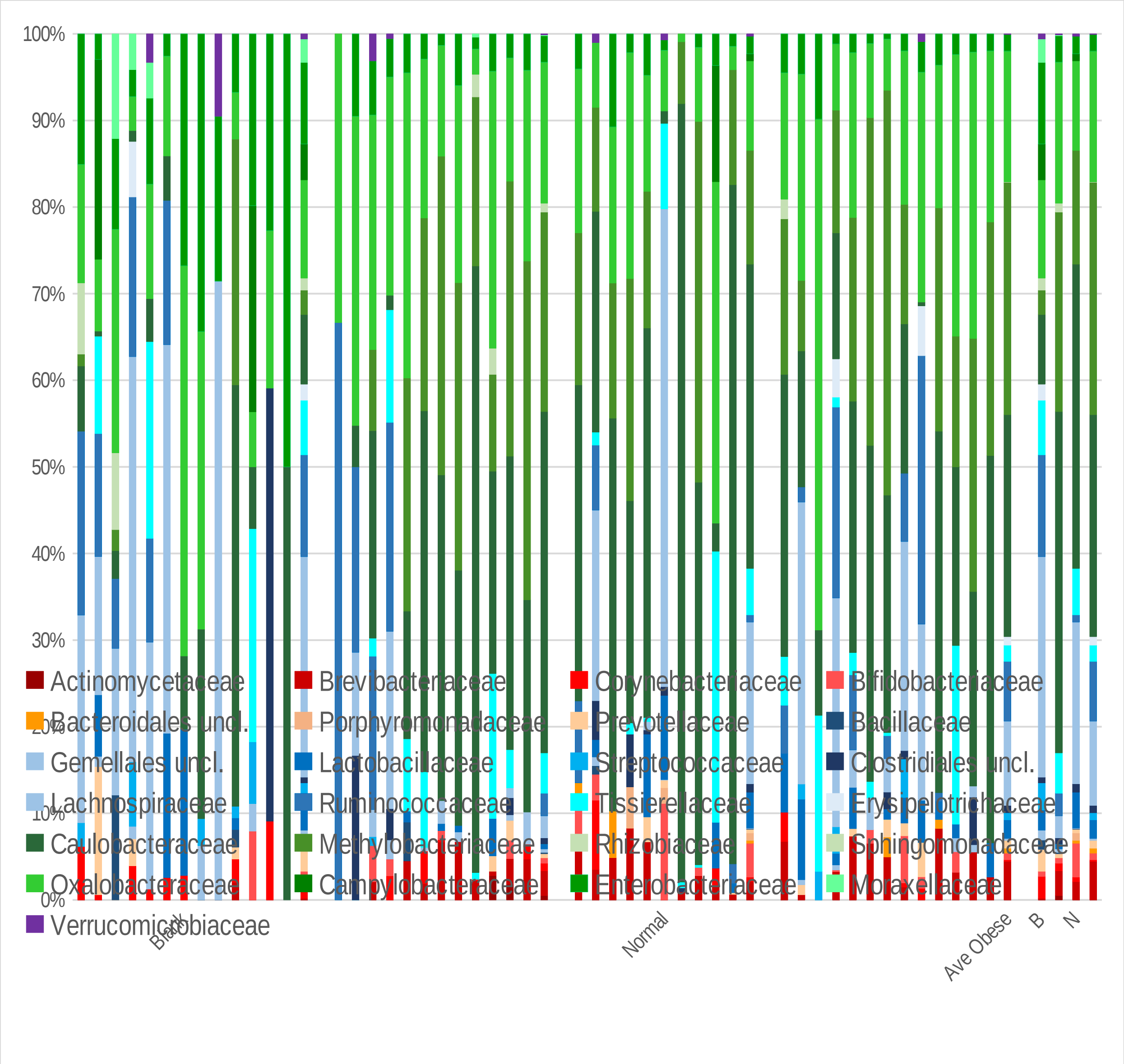
100%
90%
80%
70%
60%
50%
40%
30%
20%
10%
0%
Actinomycetaceae
Brevibacteriaceae
Corynebacteriaceae
Bifidobacteriaceae
Bacteroidales uncl.
Porphyromonadaceae
Prevotellaceae
Bacillaceae
Gemellales uncl.
Lactobacillaceae
Streptococcaceae
Clostridiales uncl.
Lachnospiraceae
Ruminococcaceae
Tissierellaceae
Erysipelotrichaceae
Caulobacteraceae
Methylobacteriaceae
Rhizobiaceae
Sphingomonadaceae
Oxalobacteraceae
Campylobacteraceae
Enterobacteriaceae
Moraxellaceae
Verrucomicrobiaceae
Normal
Ave Obese
B
N
