# Supplementary material for: Elimination of “kitome” and “splashome” contamination results in lack of detection of a unique placental microbiome
Source: BMC Microbiol. 2020 Jun 11;20:157. doi: 10.1186/s12866-020-01839-y (PMC7291729; doi:10.1186/s12866-020-01839-y)
Supplement: Supplementary file 5 [file 12866_2020_1839_MOESM5_ESM.docx]

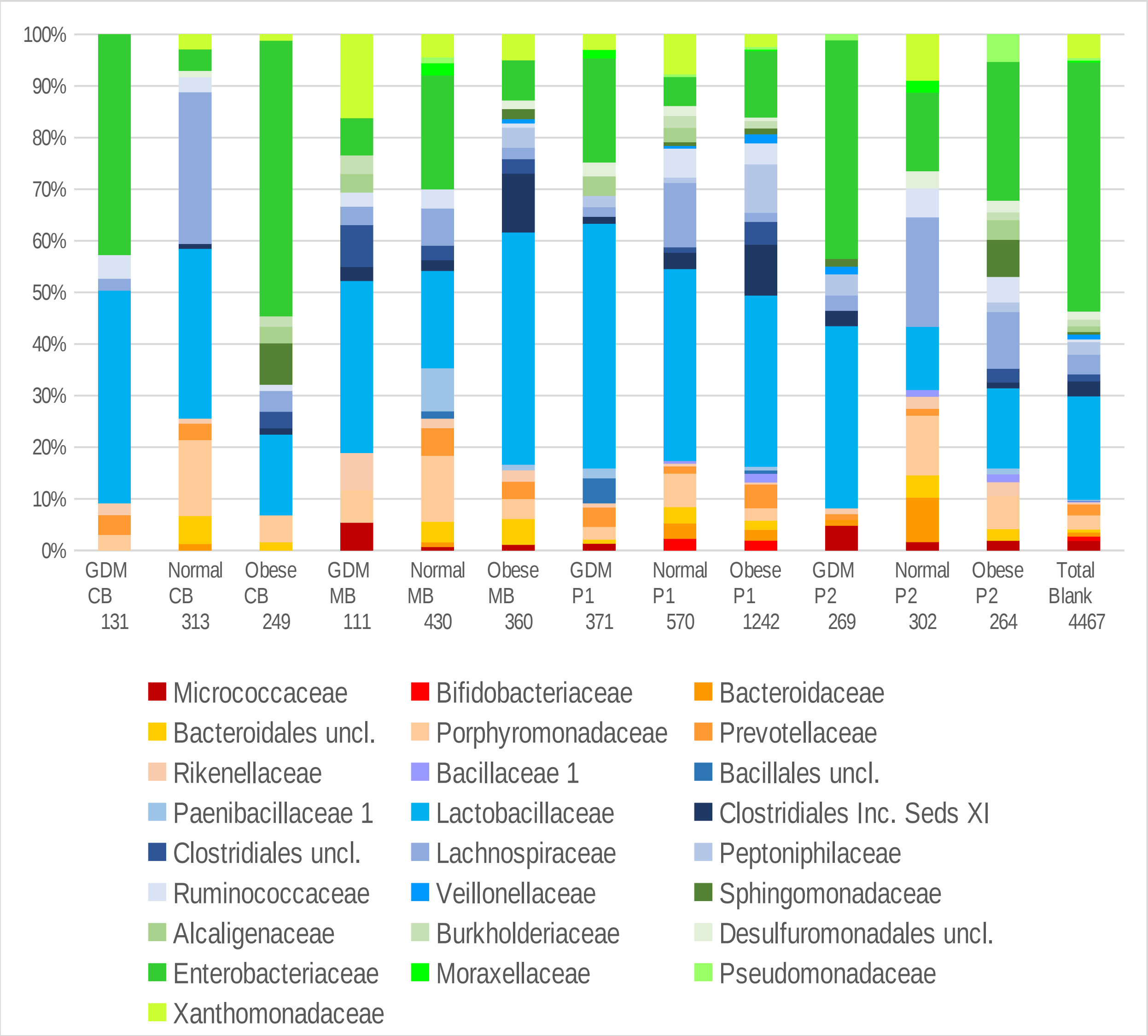
100%
90%
80%
70%
60%
50%
40%
30%
20%
10%
0%
GDM CB 131
Normal CB 313
Obese CB 249
GDM MB 111
Normal MB 430
Obese MB 360
GDM P1 371
Normal P1 570
Obese P1 1242
GDM P2 269
Normal P2 302
Obese P2 264
Total Blank 4467
Micrococcaceae
Bifidobacteriaceae
Bacteroidaceae
Bacteroidales uncl.
Porphyromonadaceae
Prevotellaceae
Rikenellaceae
Bacillaceae 1
Bacillales uncl.
Paenibacillaceae 1
Lactobacillaceae
Clostridiales Inc. Seds XI
Clostridiales uncl.
Lachnospiraceae
Peptoniphilaceae
Ruminococcaceae
Veillonellaceae
Sphingomonadaceae
Alcaligenaceae
Burkholderiaceae
Desulfuromonadales uncl.
Enterobacteriaceae
Moraxellaceae
Pseudomonadaceae
Xanthomonadaceae
